# Maintenance of magnesium homeostasis by NUF2 promotes protein synthesis and anaplastic thyroid cancer progression

**DOI:** 10.1038/s41419-024-07041-6

**Published:** 2024-09-06

**Authors:** Lisha Bao, Yingying Gong, Yulu Che, Ying Li, Tong Xu, Jinming Chen, Shanshan Wang, Zhuo Tan, Ping Huang, Zongfu Pan, Minghua Ge

**Affiliations:** 1 Otolaryngology & Head and Neck Center, Cancer Center, Department of Head and Neck Surgery, Zhejiang Provincial People’s Hospital (Affiliated People’s Hospital), Hangzhou Medical College, Hangzhou, Zhejiang China; 2 Clinical Pharmacy Center, Department of Pharmacy, Zhejiang Provincial People’s Hospital (Affiliated People’s Hospital), Hangzhou Medical College, Hangzhou, China; 3 Zhejiang Key Laboratory of Precision Medicine Research on Head & Neck Cancer, Hangzhou, China; 4 Zhejiang Provincial Clinical Research Center for malignant tumor, Hangzhou, China

**Keywords:** Endocrine cancer, Oncogenes

## Abstract

Thyroid cancer is the most frequently observed endocrine-related malignancy among which anaplastic thyroid cancer (ATC) is the most fatal subtype. The synthesis of protein is active to satisfy the rapid growth of ATC tumor, but the mechanisms regulating protein synthesis are still unknown. Our research revealed that kinetochore protein NUF2 played an essential role in protein synthesis and drove the progression of ATC. The prognosis of patients with thyroid carcinoma was positively correlated with high NUF2 expression. Depletion of NUF2 in ATC cells notably inhibited the proliferation and induced apoptosis, while overexpression of NUF2 facilitated ATC cell viability and colony formation. Deletion of NUF2 significantly suppressed the growth and metastasis of ATC in vivo. Notably, knockdown of NUF2 epigenetically inhibited the expression of magnesium transporters through reducing the abundance of H3K4me3 at promoters, thereby reduced intracellular Mg^2+^ concentration. Furthermore, we found the deletion of NUF2 or magnesium transporters significantly inhibited the protein synthesis mediated by the PI3K/Akt/mTOR pathway. In conclusion, NUF2 functions as an emerging regulator for protein synthesis by maintaining the homeostasis of intracellular Mg^2+^, which finally drives ATC progression.

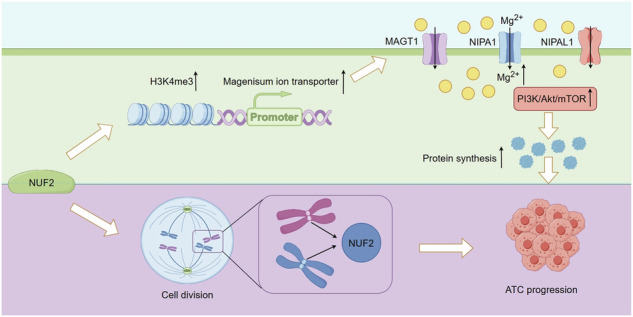

## Introduction

Thyroid cancer (TC) is the most common endocrine-related carcinoma, accounting for over 90% of all endocrine malignancies [1]. Anaplastic thyroid cancer (ATC) is the lethal subset of TC with a median survival of only six months and responsible for 40–50% of total TC-related deaths [2]. Different from other common TC subtypes, ATC grows rapidly, leading to symptoms of neck compression such as dyspnea, dysphonia, and dysphagia [3, 4]. Therefore, it is important to identify the mechanism of ATC progression.

Dysregulated protein synthesis has been shown to be one of the main characteristics of cancer [5]. To satisfy the internal oncogenic demands and external factors in the tumor microenvironment, cancer cells synthesized proteins rapidly and selectively [6]. Intracellular magnesium plays a positive role in the regulation of protein synthesis and cell proliferation [7–9]. The homeostasis of intracellular magnesium is regulated by magnesium transporters such as MAGT1, NIPA1, and NIPAL1 [10–13]. Magnesium ion is indispensable to tumor proliferation and development [14], and hypomagnesemia inhibits tumor growth and angiogenesis [15]. Studies have shown that the expression of MAGT1 impacted the progression of cancer [16, 17], and NIPA1 [18, 19] and NIPAL1 [20, 21] were related with poor prognosis. Cancer cells preferentially translate oncogenic proteins, thereby contributing to tumor growth, metastasis, and therapy resistance [22]. In ATC, the protein synthesis is also active supported by increased eukaryotic initiation factor 4E (eIF4E) and ribosome biogenesis pathway [23–26], thereby enhancing its aggressiveness and chemoresistance. Nonetheless, the exact mechanism underlying protein synthesis in ATC remains elusive.

As a constituent part of the NDC80 kinetochore complex, NUF2 is known to have a role in mitotic chromosomal alignment and the establishment of stable kinetochore-microtubule attachments [27]. The depletion of NUF2 blocked prometaphase with an active spindle checkpoint significantly [28]. NUF2 was upregulated in various kinds of cancers and regarded as a promising antitumor therapeutic target and prognostic factor [29]. Of note, several studies point out that NUF2 functions as an oncogenic regulator beyond kinetochore protein. For example, NUF2 was identified as a new cancer stem cell indicator [30]. In addition, NUF2 has been found to drive cancer development through inhibiting autophagic degradation of oncogene TFR1 [31] or affecting the transcription of HMGA2 [32]. Studies have shown that NUF2 not only affects the G2/M phase by interfering with centromere function [33], but also acts on the S phase, where protein synthesis occurs [31, 34]. However, there are few studies about the exact function of NUF2 in ATC.

In this study, the role and mechanisms of NUF2 in ATC were investigated. We found that NUF2 epigenetically regulates the transcription of magnesium transporters, thereby promoting protein synthesis through PI3K/Akt/mTOR pathway in ATC. Our findings extend the notion that NUF2 acts as an emerging regulator of magnesium homeostasis during protein synthesis and ATC progression.

## Methods

### Bioinformatic analyses

The survival curve in thyroid cancer was applied by the Kaplan-Meier plotter (http://kmplot.com/analysis). The expression level of NUF2 in TC based on nodal metastasis status was applied by UALCAN (https://ualcan.path.uab.edu/index.html). Four microarray datasets (GSE29265, GSE33630 [35], GSE65144 [36], and GSE76039 [37]) were integrated to generate a large thyroid-cancer cohort including 78 normal thyroid tissue, 69 PTCs, 17 poorly differentiated thyroid cancers (PDTCs), and 52 ATC samples as reported previously [38]. The expression of NUF2 in different groups was displayed by ggplot2 package. mTOR signaling pathway genes were obtained from Enrichr database (https://maayanlab.cloud/Enrichr) to calculate the enrichment scores by GSVA package. The relationship between the mTOR signaling score and NUF2 expression in ATC was analyzed using Spearman correlation analysis. *P* < 0.05 was considered to indicate statistical significance.

### Clinical samples

The tissue microarray including 11 normal thyroid tissues, 8 PTC, and 5 ATC were provided by Zhejiang Provincial People’s Hospital. This study was undertaken following the Declaration of Helsinki and approved by the Ethics Committee of Zhejiang Provincial People’s Hospital.

### Cell culture

The thyroid follicular epithelial cell line Nthy-ori 3-1 (ECACC, Cat# 90011609), PTC cell line BCPAP (DSMZ Cat# ACC-273), ATC cell lines 8505 C (DSMZ, Cat# ACC-219) and KHM-5M (National Collection of Authenticated Cell Cultures, Cat# SCSP-549), were cultivated in RPMI-1640 supplied with 10% fetal bovine serum (Gibco, Waltham, MA, USA) in 37 °C atmosphere containing 5% CO_2_.

### Small interfering RNA (siRNA) transfection and lentiviral infections

siRNAs targeting human NUF2 (the sense sequence was GCCGTGAAACGTATATGGAAT and the anti-sense sequence was AUUCCAUAUACGUUUCACGGC), MAGT1 (the sense sequence was GCTCATCGTTTGCGACGTT and the anti-sense sequence was AACGUCGCAAACGAUGAGC), NIPA1 (the sense sequence was GCCCAAGACATCTTGCATA and the anti-sense sequence was UAUGCAAGAUGUCUUGGGC), and NIPAL1 (the sense sequence was GCTCCAGCTTCATACTGAA and the anti-sense sequence was UUCAGAUGAAGCUGGAGC) were purchased from RiboBio (Guangzhou, China). Cells were seeded to a density of 30–40%, and siRNA and jetPRIME (Ployplus, France) were added to the culture medium. Lentiviruses for knockdown and overexpression of NUF2 and their controls were synthesized by ABM (Richmond, Canada). 4 μg/mL puromycin was used to construct stable cell lines of 8505C and BCPAP.

### Western blotting and nucleocytoplasmic separation

Total cellular protein was extracted using RIPA lysis buffer (Applygen, China, C1053-100) and quantified by Coomassie brilliant blue G-250 (Solarbio life science, China, Cat# PC0015). The extraction of nuclear and cytoplasmic proteins was performed using the nuclear-cytosol extraction kit (Applygen Technologies, China, Cat# P1200) according to the instructions. Separated by 10% SDS-PAGE gel electrophoresis, the denatured protein samples were then transferred onto the PVDF membrane (Millipore, IPFL00010). After being blocked with 5% BSA at room temperature (RT) for 1 h, the PVDF membrane was incubated with the primary antibody overnight at 4 °C and washed with Tris-buffered saline Tween (TBST) for 3 times. And then, corresponding HRP-conjugated secondary antibody was added and incubated at RT for 1 h. Images were detected by enhanced chemiluminescence. The antibodies used were listed in Table 1.

**Table 1:** The antibody used for Western blot.

Antibody	Company	Concentration
NUF2	ABclonal,Cat# A17553	1:1000
MAGT1	Proteintech Cat#17430-1-AP	1:1000
NIPA1	Santa, Cat#sc-398041	1:500
NIPAL1	Lsbio, Cat#LS-C164878	1:500
p-mTOR	Cell Signaling Technology, Cat#5536	1:1000
mTOR	Cell Signaling Technology, Cat#2983	1:1000
p-AKT	Cell Signaling Technology, Cat#4060	1:1000
AKT	Cell Signaling Technology, Cat#4691	1:1000
p-PI3K	Affinity, Cat#AF6241	1:1000
PI3K	ABclonal, Cat#A4992	1:1000
H3K4me3	ABclonal, Cat#A22146	1:1000
H3K27ac	ABclonal, Cat#A2771	1:1000
GAPDH	ABclonal, Cat# AC002	1:5000

### RNA preparation and real-time quantitative reverse transcription (RT-qPCR)

Extracted with the RNA-Quick Purification Kit (Esunbio, China), the total RNA was reversely transcribed into cDNA using the Fast All-in-One RT Kit (Esunbio, China) for qPCR. PCR amplification was performed using Super SYBR Green qPCR Master Mix (Esunbio, China). The expression levels of different genes were calculated using the 2^−ΔΔCt^ method and standardized to GAPDH. The primers used for RT-qPCR were listed in Table 2.

**Table 2:** The primers used for RT-qPCR.

Genes		primer sequence
GAPDH	Forward	5′-GCACCGTCAAGGCTGAGAAC-3′
	Reverse	5′-TGGTGAAGACGCCAGTGGA-3′
NUF2	Forward	5′-TGAAAGATACGGTCCAGAAGC-3′
	Reverse	5′-ACTGCACTTCCAACTGACATG-3′
MAGT1	Forward	5′-AAGCCCCACCGAGAAATTAC-3′
	Reverse	5′-TGAATGCACTGGAGTATCGC-3′
NIPA1	Forward	5′-CTACAGAAGAAGGGCATCGTG-3′
	Reverse	5′-CCGTGTAAGCCAGGAAGTTTC-3′
NIPAL1	Forward	5′-CAGGAGAGGCTGCAAATTTTG-3′
	Reverse	5′-CAGCCTATTTTCCCATGAATGTTC-3′

### Tissue specimens and immunohistochemistry (IHC)

Paraffin-embedded tissues were sliced and deparaffinized, and then dewaxed and hydrated with xylene and alcohol. The tissue sections were subjected to 1 mM EDTA (pH 8.0) for antigen repair. The endogenous peroxide was blocked by 0.3% hydrogen peroxide followed by treated with 3% goat serum for 30 min at RT. Primary antibodies were incubated on the slices overnight at 4 °C, followed by a 50-min RT secondary antibody incubation. Primary antibodies were listed following NUF2 (Novus, America) and p-mTOR (Proteintech, America). The immunoreaction intensity was scored as follows: negative = 0, weak = 1, moderate = 2, strong = 3. The percentage of stained cells was scored as follows: negative = 0, 0–25% = 1, 26–50% = 2, 51–75% = 3, 76–100% = 4.

### Cell viability and colony formation assay

For the cell growth assay, 3000 cells were seeded in 96-well plates. After 48 h of culture, the cells were incubated with 10 μL CCK-8 solution (FDbio, FD3788) and 90 μl PBS at 37 °C for 1 h, and then the absorbance was detected at 450 nm using the microplate reader. For the colony formation assay, KHM-5M (1000 cells) and 8505C (2000 cells) were seeded into 6-well plates and fixed after 10–14 days, and then these cells were stained with 0.2% crystal violet.

After being washed with PBS twice, the ATC cells transfected with siRNA for 48 h were incubated with Calcein AM/PI detection solution at 37 °C for 30 min protected from light according to the protocol of Cytotoxicity Assay Kit (Beyotime, China). The staining effect was observed and imaged with a fluorescence microscope.

### Flow cytometry

Annexin-V/FITC Apoptosis Kit (Multi Sciences Biotech, China) was used to assess cell apoptosis at 48 h after siRNA transfection. We resuspended the cells in 500 μL of 1× binding buffer. And then, cells which were incubated with 5 μL FITC Annexin V and 10 μL PI for 15 min at RT in the dark were detected by flow cytometry (FCM) for cell apoptosis analysis.

For cell cycle analysis, the Cell Cycle Staining Kit (Multi Sciences Biotech) was used following the manufacturer’s procedure. The collected cells were incubated for 30 min at RT in the dark with 1 mL DNA Staining Solution and 10 μL Permeabilization Solution. Then, the cell cycle was analyzed by FCM.

Cell precipitate was collected and incubated with NUF2 antibody (1:200) overnight at 4 °C on a shaker. After washing the cells with PBS, fluorescent secondary antibody (1:200) was added and incubated for 2 h at RT in the dark. And then, the cell cycle was detected by the Cell Cycle Staining Kit and FCM. Flowjo V10 software was used to analyze the expression of NUF2 in different cell cycles.

### CFSE cell proliferation assay

The cells were resuspended in serum-free medium, and 1 μL CFSE dye was added per 10 million cells to incubate the cells in a cell incubator. After 20 min, the staining was terminated by adding complete medium. After centrifugation, the cells were resuspended in the cell medium and counted, seeded in culture plates according to experimental requirements, and collected 48 h after siRNA transfection and detected by flow cytometry.

### RNA-seq analysis

Total RNA of NUF2-WT and NUF2-KD 8505C cells was isolated and purified. After quality control, Poly (A) RNA was purified and fragmented into small pieces which be synthesized into cDNA using Invitrogen SuperScript™ II Reverse Transcriptase (Thermo Fisher). Then, the ligated products were amplified with PCR. Finally, the 2 × 150 bp paired-end sequencing was carried out on illumina Novaseq™ 6000 (LC-Bio Technology, China).

Sequence quality control was performed using fastp software. Reads were mapped to the reference genome of Homo sapiens GRCh38 using HISAT2 and then assembled and calculated using StringTie (https://ccb.jhu.edu/software/stringtie). The differentially expressed genes (DEGs) were screened with fold change >2 or <0.5 and with parametric F-test comparing nested linear models (*P* < 0.05). DAVID software (https://david.ncifcrf.gov/) was used for Gene ontology (GO) enrichment analysis on DEGs. RNA-Seq data has been submitted to the GEO repository under accession number GSE272374.

### Dual-luciferase assay

293T cells were preseeded in 12-well plated with a density of 50%, and co-transfected with 500 ng luciferase reporter plasmid, and renilla luciferase reporter vector pGL-3 per well using Lipofectamine™8000 for 48 h. The experimental group was transfected with NUF2 overexpression plasmid and the control group was transfected with empty vector pcDNA3.1 for 48 h. Cells were collected and the luciferase activity was analyzed by dual-luciferase reporter gene assay kit (Beyotime, China). The firefly luciferase activity was normalized to the renilla luciferase activity that reflects expression efficiency.

### Coimmunoprecipitation (CoIP)

The protein lysates were immunoprecipitated with specific antibodies targeting against NUF2 (1:50, Novus, nbp2-99081) or IgG negative control (1:50, Beyotime, A7016) at 4 °C overnight in the rotator and then incubated with 50 μL Protein A/G Magnetic Beads pre-washed with RIPA buffer for 2 h at 4 °C in the rotator. Subsequently, the beads were collected and washed 3 times with RIPA buffer, and the immunoprecipitation compounds were separated from the beads for following western blot analysis.

### Chromatin immunoprecipitation (ChIP)

The ChIP Assay Kit (Beyotime, China, P2078) was used for the ChIP assay following the manufacturer’s protocol. In brief, glycine was used to quench ATC cells after they had been cross-linked for 10 min at 37 °C using 1% formaldehyde solution. Ultrasonication was used to cut the DNA into 200–1000 bp fragments. Then the chromatin solution was immunoprecipitated with anti-H3K4me3 or IgG antibodies (1:100). Eluted DNA was purified by DNA Purification Kit (Beyotime, China, D0033) according to the manufacturer’s procedure and analyzed the enriched genomic DNA regions by qRT-PCR. The primer sequence is listed in Table 3.

**Table 3:** The primers used for ChIP-qPCR.

Genes		primer sequence
MAGT1	Forward	5′-TGCTCAGTCGTGTCCAACTC-3′
	Reverse	5′-TGGACTGGATAGCACCCTGA-3′
NIPA1	Forward	5′-CTGTCCCCGCAGCCTAAG-3′
	Reverse	5′-AGCTGCAGTCCCCATTCC-3′
NIPAL1	Forward	5′- AGCTTCCATCCCTGCAA-3′
	Reverse	5′-CTCTTCCCCTACCACGC -3′

### Mg^2+^ measurement

Mag-Fura-4 AM (MKBio, China, MX4504-50UG) were added into cell culture media for 1 h at 37 °C following the manufacturer’s instruction. After washed with PBS 3 times, cells were incubated with 1 mM probenecid for 30 min and detected by FCM.

### Immunofluorescent staining and Giemsa staining

For immunofluorescent (IF) staining, cells were fixed with paraformaldehyde and blocked with 5% BSA. 0.2% Triton-X 100 was used for cell permeation. Then, cells were incubated with primary antibodies against NUF2 (1:200) and α-tublin (1:200) at 4 °C overnight followed by secondary fluorescent antibodies protected from light for 2 h. And then, cells were washed with PBS for three times and incubated with DAPI for 15 min. Imaging was carried out using a fluorescence microscope.

Giemsa staining experiments were performed according to the instructions of the rapid Giemsa staining kit (BBI Life Sciences Corporation, China, E607314-0001). The cells were fixed with 4% paraformaldehyde for 10 min at RT and then incubated with the Giemsa staining working solution (solution I: solution II = 1:9) for 30 min at RT. After washed with PBS for 3 times, cells were observed and recorded under a microscope.

### Animal models

Fertilized eggs from wild-type AB strains of zebrafish were cultivated in E3 embryo medium containing 0.2 mM 1-phenyl-2-thio-urea (PTU; Sigma, Germany) at 28 °C. WT or NUF2-KD ATC cells (8505C and KHM-5M) were labeled with 5 µg/mL DiI for 15–30 min at 37 °C in the dark. Resuspended cells were injected into the perivitelline space of zebrafish at 48 h-post-fertilization (hpf). Zebrafishes were imaged under the fluorescence microscope at 0 and 3 days after microinjection, respectively. Re.Size = Fluorescence area at 3 days/Fluorescence area at 0 days.

Four-week-old female BALB/c nude mice were randomly assigned (*n* = 6/group) to established mouse models. WT or NUF2-KD 8505 C cells (4 × 10^6^) were collected and resuspended in 100 μL PBS supplemented with 10% Matrigel (v/v) and then injected subcutaneously into nude mice. The body weight and tumor volume of the tumor-bearing mice were measured and recorded every 2–3 days. The tumor volumes were calculated as follows: Tumor volume = length × width^2^/2. At the endpoint, the mice were sacrificed, and tumor tissues were excised, weighted and photographed.

To establish ATC orthotopic models, luciferase-labeled 8505 C cells were resuspended in PBS (1 × 10^7^/mL), and 10 μL of the cell suspension was injected into the thyroid lobe of 6-week-old nude mice by neck surgery. Mice were anesthetized with isoflurane and injected with 100 µl d-luciferin (3 mg/mouse) for bioluminescent imaging. As for the ATC pulmonary metastasis, luciferase-labeled 8505 C cells (1 × 10^6^) was suspended in 100 µL PBS and injected into the tail vein of nude mice. The bioluminescent imaging was performed weekly, for 4 weeks.

The animal protocols were approved by the Animal Welfare Committee of Zhejiang Provincial People’s Hospital (Approved number: 20210510011).

### Protein synthesis assay

The Global Protein Synthesis Assay Kit (Abcam, America, ab235634) was used to detect the protein synthesis efficiency. The cells were incubated with the protein label for 0.5–2 h at 37 °C. Cells were fixed by Fixation solution for 15 min at RT in the dark and then incubated with 1 × Reaction cocktail for 30 min at RT in the dark. After washing and proceeding with DNA staining, cells were analyzed by fluorescence microscope and FCM.

### Data processing and statistical analysis

Statistical significance between the two groups was calculated by two-tailed Student’s t-test. Two-sided *P* < 0.05 was considered statistically significant. (*P < 0.05, **P < 0.01, ***P < 0.001). Graphical abstract was drawn by Figdraw.

## Results

### NUF2 was overexpressed in ATC and correlated with poor prognosis

**Fig. 1:**
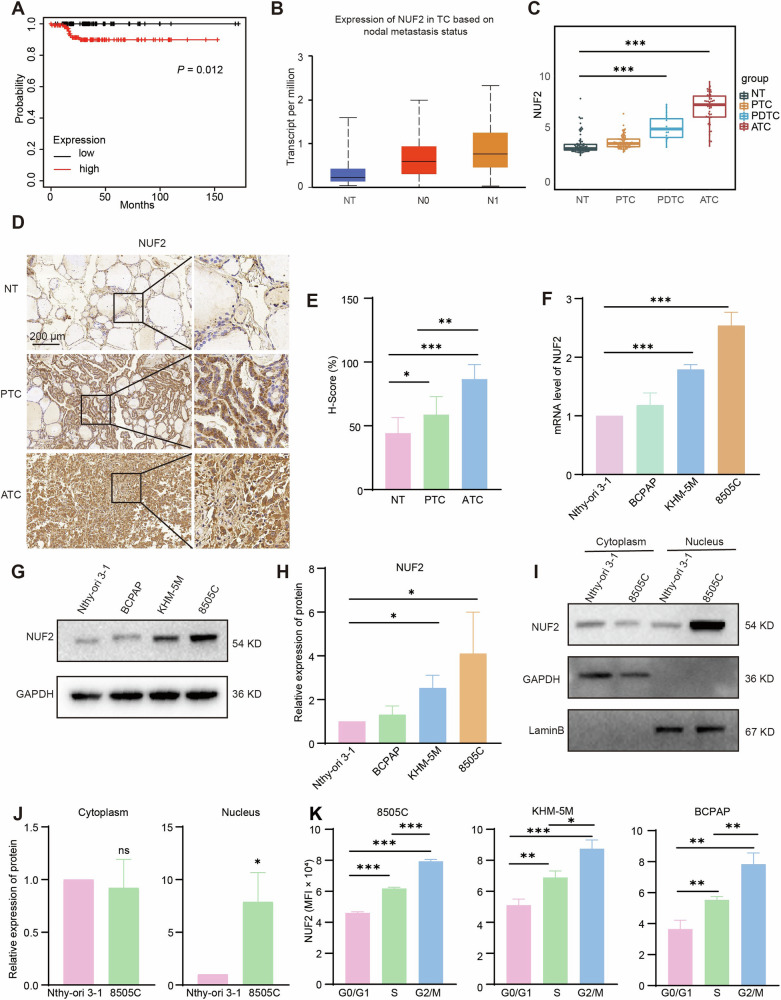
Expression of NUF2 in ATC and clinical relevance. **A** The relevance of NUF2 expression and relapse-free survival (RFS) in patients with thyroid cancer (TC). **B** Expression of NUF2 in TC subgroups based on nodal metastasis status. **C** The expression of NUF2 in NT, PTC, PDTC, and ATC based on four microarray datasets from GEO database. **D**, **E** IHC staining of NUF2 in NT and ATC tissues. **F**–**H** The mRNA and protein levels of NUF2 in different thyroid cancer cell lines. **I**, **J** The expression of NUF2 in cytoplasm and nucleus. **K** The expression of NUF2 in different cell cycle phases. Data are presented as the mean ± SD. *P < 0.05, **P < 0.01, ***P < 0.001.

According to the survival analysis and expression level analysis based on nodal metastasis status, high expression of NUF2 was correlated with poor prognosis and positively related with nodal metastasis status (Fig. 1A, B). Besides, NUF2 was overexpressed in ATC according to the thyroid-cancer cohort reported previously [39] (Fig. 1C). Further IHC staining of tissue microarray confirmed that NUF2 expressed at a higher level in ATC tissues (H-score = 86.73) than normal thyroid tissues (H-score = 44.27) (Fig. 1D, E). The mRNA and protein expression levels of NUF2 were also upregulated in the ATC cell lines, KHM-5M and 8505 C, compared to the thyroid follicular epithelial cell line, Nthy-ori 3-1, and the PTC cell line, BCPAP (Fig. 1F–H). Collectively, these data support that NUF2 was upregulated in thyroid cancer and and its level is positively correlated with poor prognosis. Subsequently, we explored the expression of NUF2 in different cell localizations and cell cycle phases. The result of nucleocytoplasmic separation showed that nuclear NUF2 was remarkably increased in ATC than that of Nthy-ori 3-1 (Fig. 1I–J). Although NUF2 was known as a component of kinetochore complex, it gradually increased along the cell cycle, and expressed most abundantly in G2/M phase (Fig. 1K), indicating a complicated role of NUF2 in ATC.

### NUF2 is associated with proliferation and apoptosis in ATC cells

**Fig. 2:**
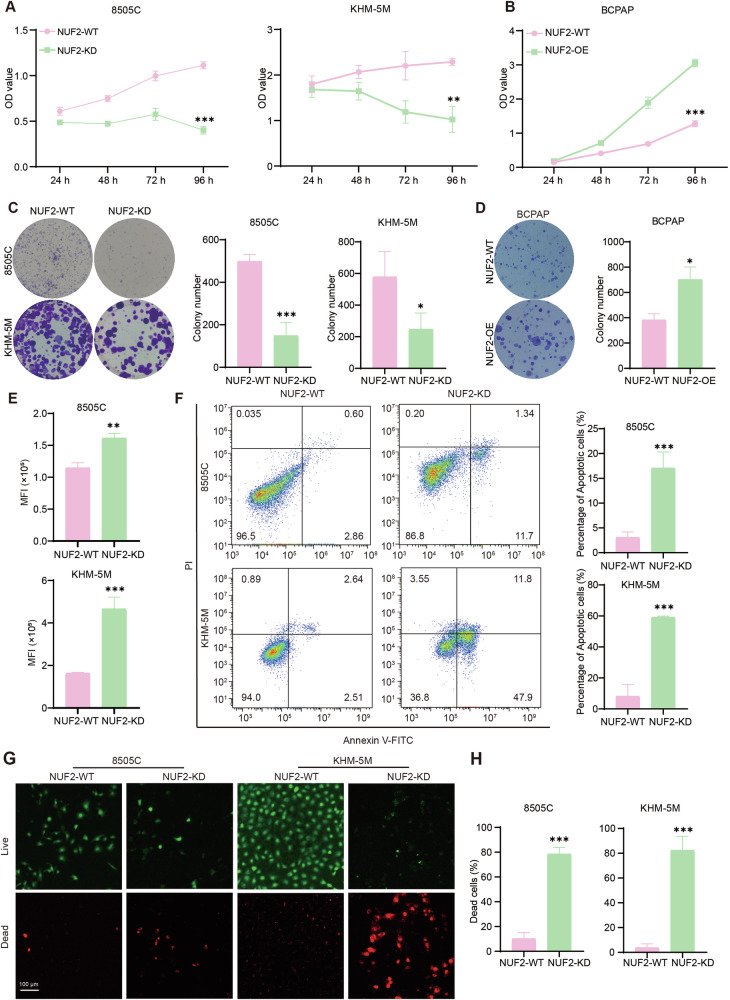
Expression of NUF2 is associated with proliferation and apoptosis in ATC cells. **A** In vitro growth curves of ATC cells transfected with siNUF2. **B** In vitro growth curves of BCPAP cells after NUF2 overexpression. **C** Colony formation of ATC cells transfected with siNUF2. **D** Colony formation of BCPAP cells with NUF2 overexpression. **E** The cell division was determined by CFSE after NUF2 knockdown by siRNA. **F** Apoptosis of ATC cells 48 h after NUF2 knockdown by siRNA. **G**, **H** Cell viability of ATC cells transfected with siNUF2 (Live cells: Calcein-AM, Dead cells: PI). Data are presented as the mean ± SD. *P < 0.05, **P < 0.01, ***P < 0.001.

In order to understand the function of NUF2 in thyroid cancer, we then evaluated the effects of NUF2 on ATC cell proliferation and apoptosis in vitro. The transfected efficiency of siRNA-NUF2 (Fig. S1A, B) and NUF2 overexpression lentiviruses (Fig. S1C, D) was validated using qRT-PCR and western blot. The deletion of NUF2-induced karyoklasis and increased the separation error percentage. NUF2-KD ATC cells were accumulated in G2/M phase while NUF2 overexpression reduced the percentage BCPAP cells in G2/M phase (Fig. S2). NUF2 knockdown in ATC cells significantly attenuated proliferation potency (Fig. 2A). In contrast, overexpression of NUF2 notably increased cell viability (Fig. 2B). Colony formation ability was inhibited in NUF2-KD ATC cell lines and increased in NUF2-OE PTC cell lines (Fig. 2C, D). Cell division determined by CFSE confirmed that loss of NUF2 hindered ATC proliferation (Fig. 2E). Furthermore, knockdown of NUF2 dramatically induced apoptosis in ATC cell lines (Fig. 2F). Live/Dead assay was then used to evaluate the effect of NUF2 knockdown on ATC viability. The percentage of dead cells labeled with PI (red) was increased after knocking down NUF2 compared with that of control (Fig. 2G, H). These results suggested that NUF2 is essential for the growth and survival of thyroid cancer cells.

### NUF2 promoted ATC tumor growth and metastasis in vivo

**Fig. 3:**
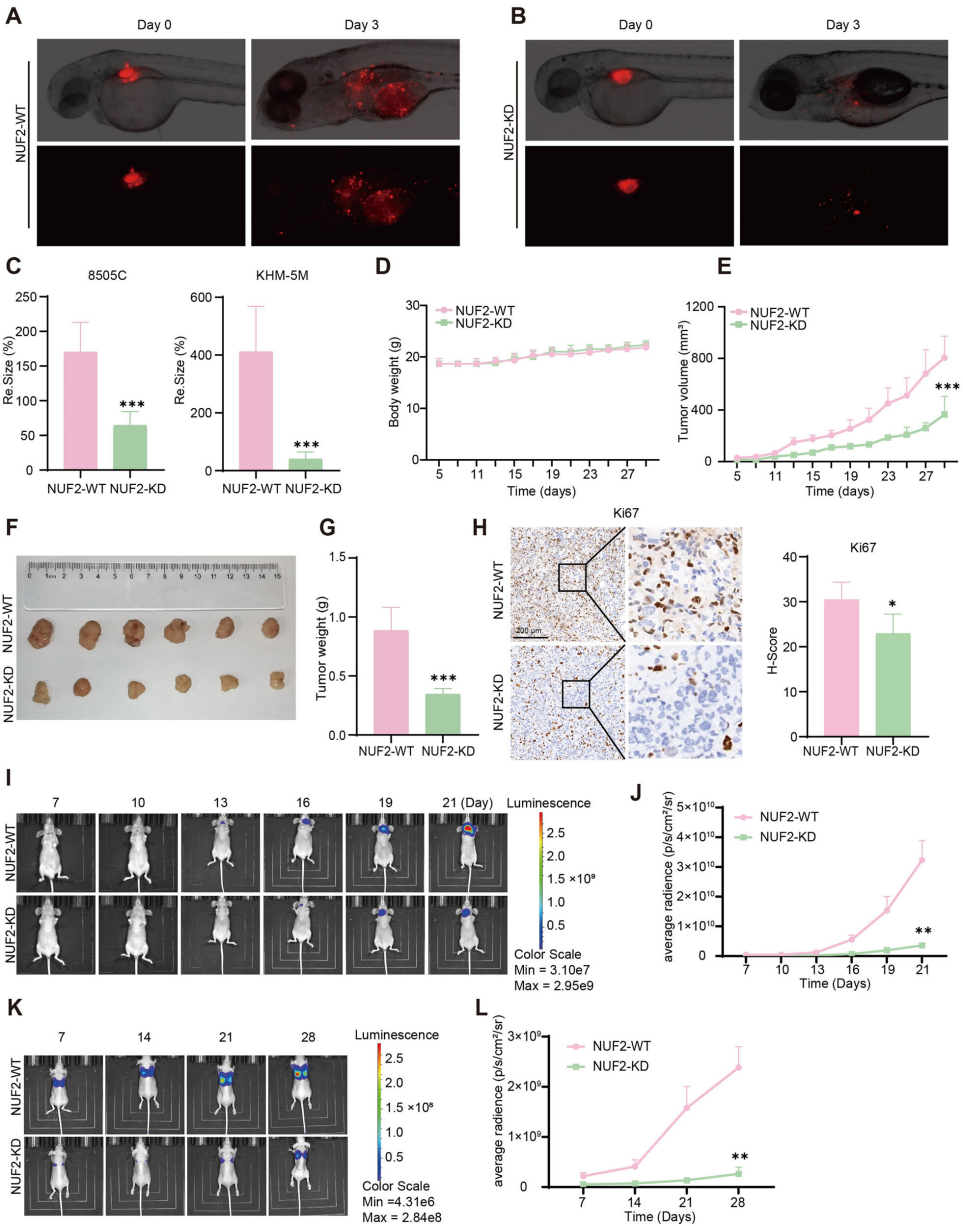
NUF2 promoted ATC tumor growth and metastasis in vivo*.* **A**–**C** Tumor growth from zebrafish-CDX model in control and NUF2-KD groups. **D** Weight growth curve of nude mice. **E, F** The ATC xenograft tumor volume and growth curve in nude mice. **G** Tumor weight of control and NUF2-KD groups. **H** IHC staining of Ki67 in mouse xenografts. **I** Representative images showing luciferase activity in orthotopic ATC tumor xenograft. **J** Luciferase activity curve of orthotopic ATC tumor model. **K, L** The tumor metastasis in nude mice. n = 6 mice per group. Data are presented as the mean ± SEM. *P < 0.05, **P < 0.01, ***P < 0.001.

To investigate the effect of NUF2 on tumor growth in vivo, we implanted control or NUF2-knockdown ATC cells into zebrafish and nude mice. In zebrafish-CDX model, the fluorescence size of ATC xenograft in NUF2-knockdown group was significantly decreased after 3 days (Fig. 3A, B). The fluorescence size of 8505 C and KHM-5M decreased by 2.6 times and 10.2 times compared with those of control, respectively (Fig. 3C). Consistently, growth of ATC xenografts derived from nude mice were remarkably inhibited in NUF2-knockdown group compared with control (Fig. 3D, E). Both tumor volume and tumor weight were dramatically attenuated after deletion of NUF2 (Fig. 3F, G). The IHC staining of mice tumor tissues confirmed that NUF2 knockdown decreased the expression of proliferative nuclear marker Ki67 (Fig. 3H) [40]. In addition, we established orthotopic ATC tumor model in nude mice and found the tumor growth was significantly inhibited by effect of NUF2 knockdown (Fig. 3I, J). After NUF2-KD and control 8505C cells were injected into the tail vein nude mice, we collected images and calculated luciferase activity to dynamically evaluate the lung metastases. The result showed that NUF2 knockdown remarkably decreased the metastatic ability of ATC cells (Fig. 3K, L). Collectively, these results showed that NUF2 promoted the progression and metastasis of ATC in vivo.

### NUF2 was required for the magnesium ion transport in ATC

**Fig. 4:**
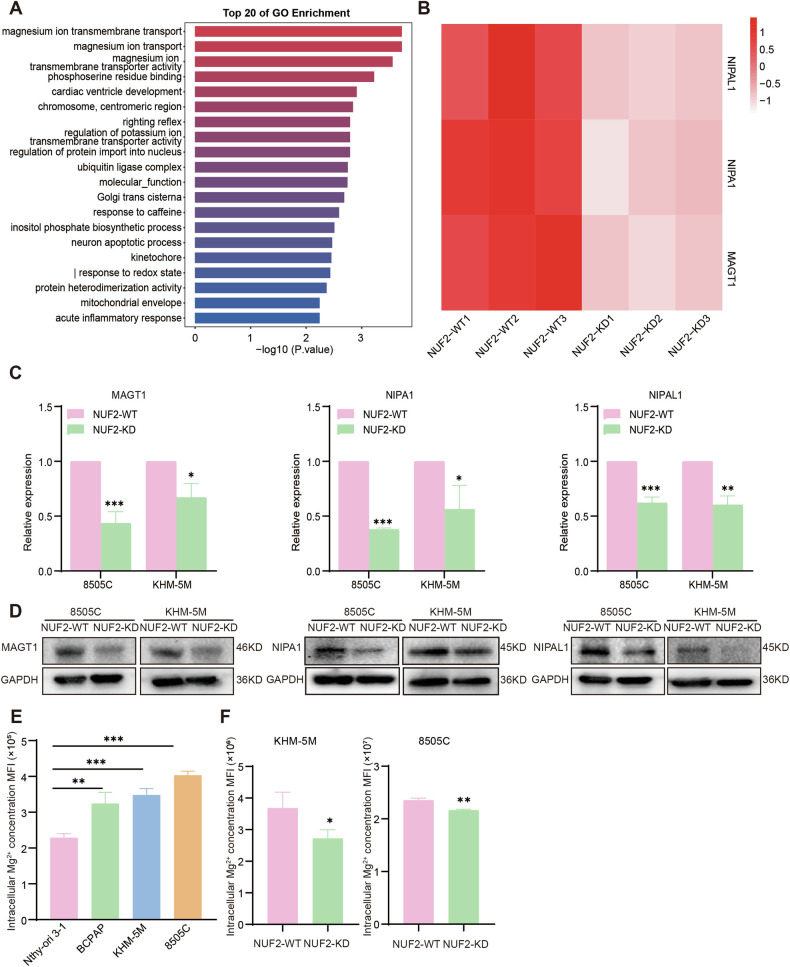
NUF2 knockdown inhibited the magnesium ion transport pathway in ATC. **A** Top 20 of GO enrichment according to the data of RNA-sequencing in 8505C cells which were transfected with siRNA-NUF2 and siRNA-NC, respectively**. B** The expression levels of genes in magnesium ion transport pathway. **C**, **D** The mRNA and protein levels of MAGT1, NIPA1, and NIPAL1 in NUF2-WT and NUF2-KD ATC cells. **E** The intracellular Mg^2+^ concentration of Nthy-ori 3-1, BCPAP, KHM-5M, and 8505C cell lines. **F** The intracellular Mg^2+^ concentration of NUF2-WT and NUF2-KD ATC cells (8505C, KHM-5M). Data are presented as the mean ± SD. *P < 0.05, **P < 0.01, ***P < 0.001.

To explore the underlying mechanism of NUF2 in ATC progression, RNA-sequencing (RNA-seq) analysis was performed to compare relative changes of gene expression in siNUF2 and control cells. Then, we performed GO analysis on the differentially expressed genes and listed the top 20 enriched pathways in Fig. 4A. Notably, magnesium ion transport pathways ranked in the top 3 (Fig. 4A). The magnesium ion transport pathways consist of three typical magnesium ion transport-related genes (MAGT1, NIPA1, NIPAL1), and they were down-regulated in NUF2-knockdown ATC cells (Fig. 4B). Consistently, the results of qRT-PCR and western blot proved that NUF2 knockdown in ATC cell lines inhibited the mRNA and protein expression level of MAGT1, NIPA1 and NIPAL1 (Fig. 4C, D). Compared with Nthy-ori 3-1, the thyroid cancer cell lines especially ATC cell lines showed a higher level of intracellular magnesium ion concentration (Fig. 4E). The deletion of NUF2 decreased the intracellular magnesium ion concentration in ATC cell lines indicating a potential of NUF2 in regulating magnesium transportation (Fig. 4F). In conclusion, NUF2 was related with the magnesium ion transport process in ATC cells.

### NUF2 promoted cell proliferation by regulating magnesium transporters

**Fig. 5:**
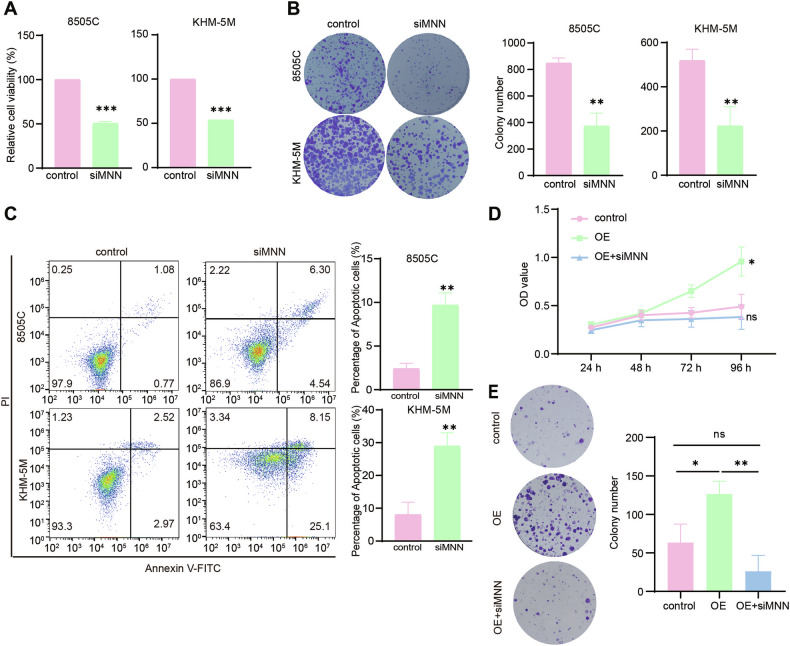
NUF2 promoted cell proliferation by regulating magnesium transporters. **A** Cell viability of 8505C and KHM-5M cells transfected with siMNN (MAGT1 + NIPA1 + NIPAL1) or siRNA-NC. **B** Colony formation of control or siMNN 8505 C and KHM-5M cells. **C** Apoptosis of control and siMNN ATC cells. Growth curve (**D**) and colony formation (**E**) of BCPAP cells with NUF2 overexpression and siMNN. Data are presented as the mean ± SD. *P < 0.05, **P < 0.01, ***P < 0.001.

To uncover the role of magnesium transporters in ATC progression, we knock downed MNN (MAGT1, NIPA1, and NIPAL1) in ATC cells and verified the knockdown efficiency (Fig. S1E, F). Loss of MAGT1, NIPA1, and NIPAL1 showed significantly decreased cell viability and colony formation ability in ATC cells (Fig. 5A, B). In addition, the deletion of magnesium transporters increased the percentage of apoptotic cells (Fig. 5C). Moreover, we further investigated whether MNN is a key mediator of the oncogenic effect of NUF2 in ATC. CCK-8 assay presented that knockdown of MNN abolished NUF2-induced promotion of tumor growth (Fig. 5D). The result of colony formation showed that depletion of MNN abolished NUF2-induced promotion of colony formation ability (Fig. 5E). In general, these results showed that the upregulation of magnesium transporters was partly responsible for NUF2-mediated tumor progression in ATC.

### NUF2 regulates the transcription of magnesium ion transporters by H3K4me3

**Fig. 6:**
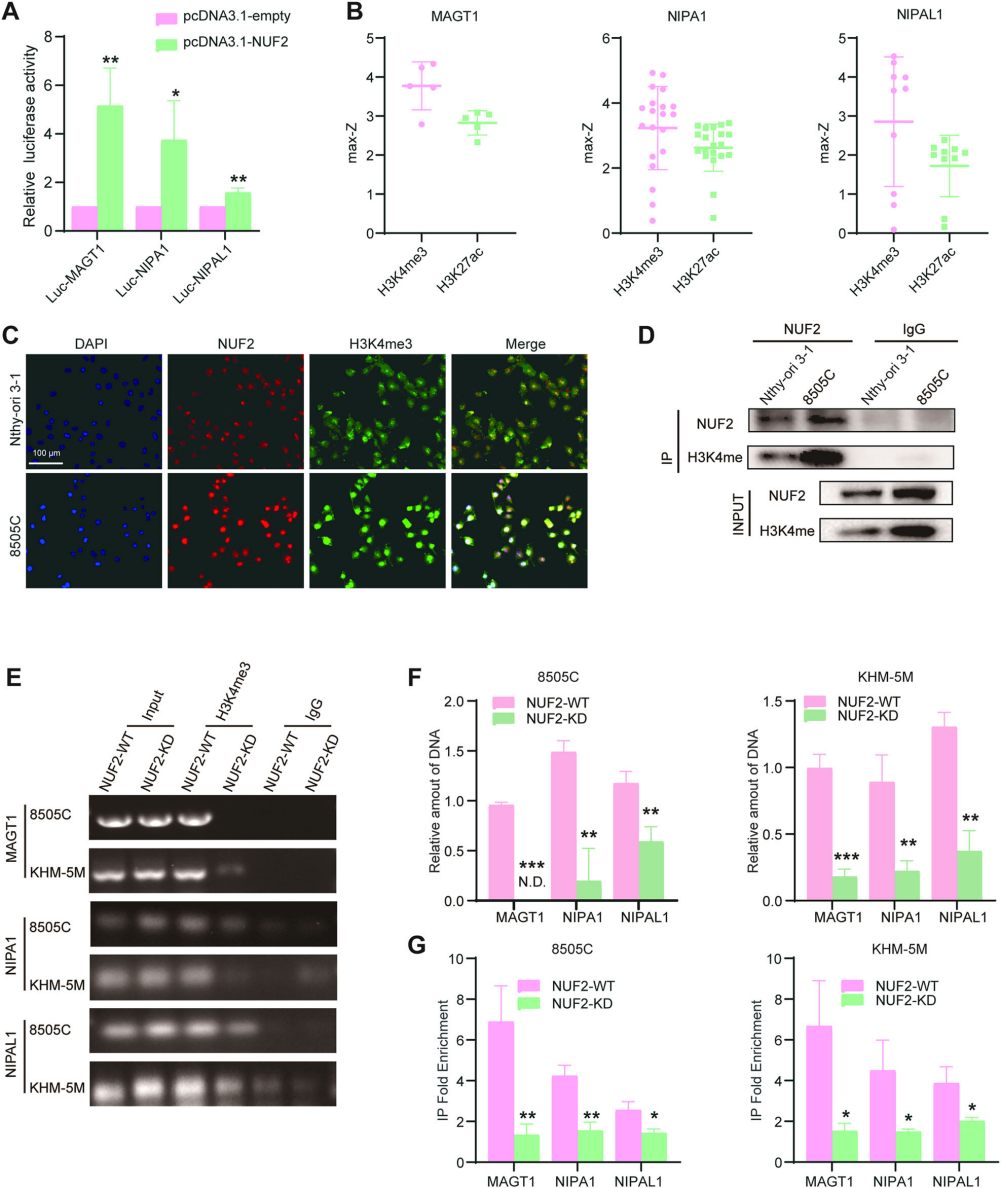
NUF2 regulate the transcription of magnesium ion transporters by H3K4me3. **A** Relative luciferase activity in 293T cells treated with pcDNA3.1 empty vector and NUF2 plasmids. **B** Max-Z score of H3K4me3 and H3K27ac in the thyroid glands of four patients. **C** Double immunofluorescence for NUF2 and H3K4me3 in 8505 C and Nthy-ori 3-1 cells. **D** Coimmunoprecipitation (CoIP) assay of NUF2, H3K4me3 and H3K27ac. **E**–**G** The abundance of H3K4me3 at the MAGT1, NIPA1 and NIPAL1 promoters in ATC cell lines was analyzed by chromatin immunoprecipitation. Data are presented as the mean ± SD. *P < 0.05, **P < 0.01, ***P < 0.001.

Next, we explored the mechanism underlying magnesium signaling regulated by NUF2. Subsequently, MAGT1, NIPA1 and NIPAL1 promoter-luciferase reporter plasmids were constructed and transfected into 293T cells individually. The result of dual-luciferase reporter (DLR) gene assays showed that NUF2 overexpression significantly elevated the transcriptional activity of MNN (Fig. 6A). Considering that histone modification is essential for modulating the transcription of downstream genes, we sought to assess the possible histone modification at the promotor of MNN in thyroid glands. Based on the prediction of the SCREEN database (https://screen.encodeproject.org/), we found that the mean max-Z score of H3K4me3 was higher than H3K27ac at the promoter of MNN in four thyroid tissues, indicating that H3K4me3 might regulate the transcription of MNN (Fig. 6B). Immunofluorescence staining showed that H3K4me3 and NUF2 were highly expressed in 8505 C and they were expressed in the same site of nuclear (Fig. 6C). In addition, CoIP assay also demonstrated the interaction between H3K4me3 and NUF2 (Fig. 6D). According to the prediction of the SCREEN database, we designed and synthesized the corresponding primers (Table 3) for ChIP-qPCR. The result showed that NUF2 knockdown significantly inhibited H3K4me3 occupancy on the MNN promoter (Fig. 6E–G). Taken together, these findings demonstrated that NUF2 regulates the transcription of magnesium transporters by H3K4me3.

### NUF2-mediating magnesium homeostasis affected the efficiency of protein synthesis

**Fig. 7:**
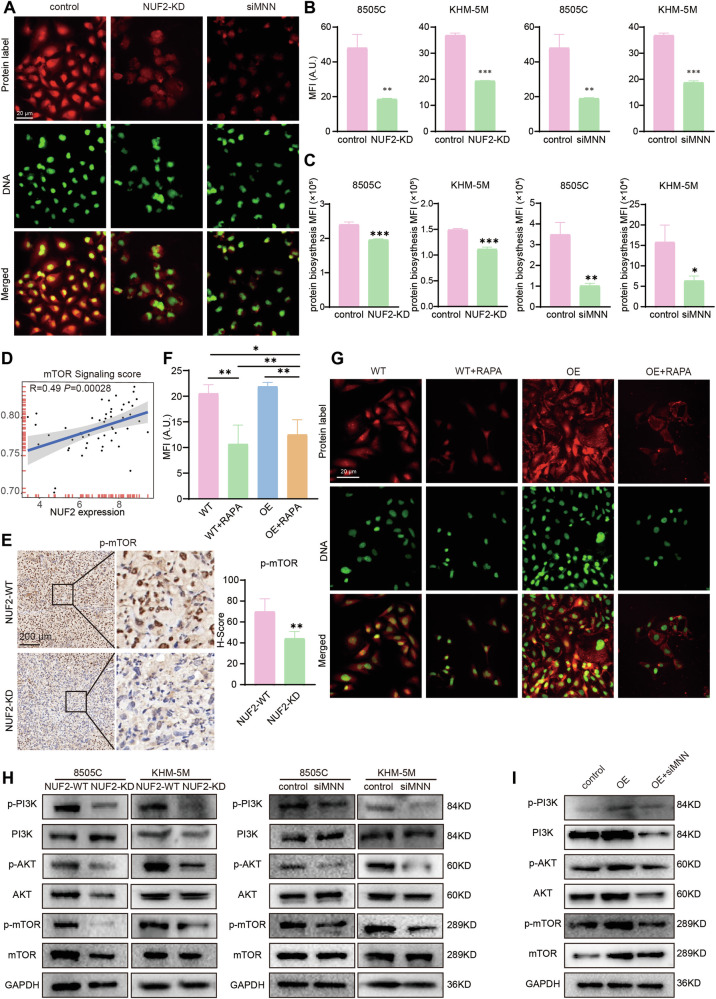
NUF2-mediating magnesium homeostasis affected the efficiency of protein synthesis by PI3K/Akt/mTOR pathway. **A**–**C** Nascent proteins in ATC cells transfected with siNUF2 or siMNN. **D** Spearman correlation analysis of mTOR signaling score and NUF2 expression in ATC samples. **E** IHC staining of p-mTOR in mouse xenografts. **F**, **G** Protein synthesis in WT and NUF2-OE BCPAP cells treated with mTOR inhibitor RAPA. **H** Western blot analysis of PI3K/Akt/mTOR signaling followed by NUF2 knockdown or siMNN. **I** Western blot analysis of PI3K/Akt/mTOR signaling followed by NUF2 overexpression and siMNN. Data are presented as the mean ± SD. *P < 0.05, **P < 0.01, ***P < 0.001.

It has been demonstrated that intracellular Mg^2+^ plays a central role in the regulation of DNA and protein synthesis [7]. Therefore, we detected the nascent proteins in cells used a protein synthesis assay kit and the protein synthesis in NUF-KD ATC cells were significantly inhibited. Consistently, there were fewer nascent proteins in cells that simultaneously knocked down MAGT1, NIPA1, and NIPAL1 (Fig. 7A–C). It has been verified that the PI3K/Akt/mTOR pathway participated in the protein synthesis, and Mg^2+^ could regulate the activation of the PI3K/Akt/mTOR pathway [41]. Thus, we try to explore whether NUF2-mediating magnesium homeostasis affected the efficiency of protein synthesis by PI3K/Akt/mTOR pathway. The Spearman correlation analysis demonstrated the positive correlation between the expression level of NUF2 and mTOR signaling in ATC samples (Fig. 7D). Compared with NUF2-WT group, the expression level of p-mTOR was lower in NUF2-KD mouse xenograft tissues (Fig. 7E). Furthermore, we tested the IC50 of mTOR inhibitor RAPA (Fig. S3) and RAPA at the concentration of IC50 was added to BCPAP cells. The result showed that the increased protein synthesis induced by NUF2 overexpression could be partially weakened by mTOR inhibition (Fig. 7F, G). The knockdown of NUF2 or MNN inhibited the phosphorylation of PI3K/Akt/mTOR in ATC cells (Fig. 7H). Moreover, we demonstrated the rescue effects of MNN for the upregulation of PI3K/Akt/mTOR pathway induced by NUF2. The promoting effect of NUF2 overexpression on PI3K/Akt/mTOR pathways could be in part reversed by MNN knockdown (Fig. 7I). These experiments indicated NUF2-mediating magnesium homeostasis affected PI3K/Akt/mTOR pathway, then influencing the efficiency of protein synthesis.

## Discussion

ATC is the most fatal subset of TC [1] and is characterized by a rapid growth rate which results in neck compression symptoms and death [3, 4]. NUF2 has been reported upregulated in various kinds of cancers including colorectal, gastric, breast, renal, and ovarian cancer [29, 30, 32, 42, 43]. Deletion of NUF2 can significantly inhibit the tumorigenesis and progression of cancers in vitro and in vivo [44–46]. However, there were little study on the function of NUF2 in ATC. In our study, we confirmed that NUF2 is a crucial regulator for protein synthesis in ATC. NUF2 transcriptionally regulates the expression of magnesium transporters by affecting H3K4me3 abundance at the promoter, thereby mediating magnesium homeostasis and promoting protein synthesis, which finally drives ATC progression.

NUF2 has been widely known as a constituent part of the NDC80 kinetochore complex and participated in maintaining the stability of kinetochore-microtubule attachments and chromosome alignment in mitosis. Consistent with its function, NUF2 was widely considered to play a pro-tumor role by regulating cell division and DNA replication [29, 34]. We found that NUF2 knockdown induced karyoklasis and increased the separation error percentage in ATC. High rates of separation error rates led to cell death and tumor suppression [47]. Recent research also revealed that NUF2 may drive cancer progression through other mechanisms. For example, NUF2 was identified as a new cancer stem cell indicator in breast cancer [30]. In ovarian cancer, NUF2 contributed to tumor initiation and development through PI3K-AKT and MAPK signaling axes mediated by ERBB3 [42, 43]. In addition, NUF2 can also facilitate the development of cholangiocarcinoma through p38/MAPK signaling by suppressing the p62 binding of TFR1 and affecting its autophagic degradation [31]. NUF2 acts as an oncogene in renal cancer by affecting the expression of HMGA2 by KDM2A-mediated H3K36me2 demethylation [32]. Our finding showed that NUF2 was required for the survival and tumor growth of ATC, which was dependent on NUF2-mediated magnesium ion homeostasis. 8505C cell line was from an ATC tissue while KHM-5M cell line was from a pleural metastasis. The differences in genetic background between the 8505C cell line and the KHM-5M cell line may contribute to their difference in cell proliferation and apoptosis. In our research, 8505C cell lines showed higher expression level of NUF2 than KHM-5M. Compared with 8505C cell line, knockdown of NUF2 inhibited the proliferation and induced cell apoptosis in vitro more dramatically in KHM-5M cell line. In the CDX model of zebrafish, knockdown of NUF2 resulted in more significant tumor suppression in KHM-5M cells than in 8505C cells.

Magnesium is an essential ion that plays an indispensable role in various kinds of cellular processes including cell proliferation, migration, and apoptosis [48]. Magnesium was normally regarded as ‘second messenger’ to regulate multiple reactions involving the response to growth factors that eventually lead to cell division [49]. The efficiency of protein and DNA synthesis increased sharply with small increases of intracellular Mg^2+^ in the physiological range [8]. Hypomagnesemia has been shown to prevent angiogenesis and tumor growth [15]. The intracellular magnesium concentration was regulated by some magnesium transporters including NIPA1, NIPAL1, and MAGT1 [10–13]. These magnesium transporter genes also be related with cancer progression. For example, NIPA1 [18, 19] and NIPAL1 [20, 21] were related with poor prognosis of cancers, and MAGT1 knockdown inhibited tumor progression [16, 17]. The result of our study has demonstrated that NUF2 is involved in the magnesium ion transport in ATC. Furthermore, we found that NUF2 regulated the transcription of magnesium transporters by increasing the abundance of H3K4me3 at the promoter, which is regarded as one of the most recognized epigenetic marks of active transcription [50].

The dysregulation of protein synthesis is a distinctive feature of the cancer cells. The rapid proliferation of cancers requires the synthesis of large amounts of proteins in cells [6]. The active protein synthesis was supported by increased eukaryotic initiation factor and ribosome biogenesis [23–26], thereby promoting the aggressiveness and chemoresistance of ATC. However, the exact mechanism underlying protein synthesis in ATC still remains unknown. The PI3K/Akt/mTOR signaling is essential for the regulation of many basic cellular processes including protein synthesis and the dysregulation of PI3K/Akt/mTOR signaling is associated with cancer progression [41]. It has been reported that magnesium influx mediated by TRPM7-regulated signaling throughout the PI3K/Akt/mTOR pathway [51, 52]. Besides, studies have shown that mTOR may be a magnesium-sensitive crucial regulator of protein synthesis related with proliferative signaling [49]. This evidence supports the hypothesis that magnesium ions influence protein synthesis by PI3K/AKT/mTOR pathway. Therefore, we try to establish a link between NUF2-mediating magnesium homeostasis and protein synthesis. It has been shown in our study that the knockdown of NUF2 or magnesium transporters significantly inhibited PI3K/Akt/mTOR signaling and protein synthesis efficiency.

In conclusion, our study showed that NUF2 epigenetically promotes the transcription of magnesium transporters and facilitates Mg^2+^-dependent protein synthesis by activating PI3K/Akt/mTOR pathway, thereby driving the malignant phenotype of ATC.

## Supplementary information

Supplementary Material

Orinigal Western blot

## Data Availability

The data supporting the conclusions of this paper have been provided in this paper and GEO database. In addition, all data for this study are available from the corresponding author upon reasonable request.
