# Correction: Maintenance of magnesium homeostasis by NUF2 promotes protein synthesis and anaplastic thyroid cancer progression

**DOI:** 10.1038/s41419-024-07174-8

**Published:** 2024-11-18

**Authors:** Lisha Bao, Yingying Gong, Yulu Che, Ying Li, Tong Xu, Jinming Chen, Shanshan Wang, Zhuo Tan, Ping Huang, Zongfu Pan, Minghua Ge

**Affiliations:** 1 Otolaryngology & Head and Neck Center, Cancer Center, Department of Head and Neck Surgery, Zhejiang Provincial People’s Hospital (Affiliated People’s Hospital), Hangzhou Medical College, Hangzhou, Zhejiang China; 2 Clinical Pharmacy Center, Department of Pharmacy, Zhejiang Provincial People’s Hospital (Affiliated People’s Hospital), Hangzhou Medical College, Hangzhou, China; 3 Zhejiang Key Laboratory of Precision Medicine Research on Head & Neck Cancer, Hangzhou, China; 4 Zhejiang Provincial Clinical Research Center for malignant tumor, Hangzhou, China

**Keywords:** Endocrine cancer, Oncogenes

Correction to: *Cell Death and Disease* 10.1038/s41419-024-07041-6, published online 06 September 2024

In this article, Fig. S2A has been replaced. There is a wrong picture (region of interest) of Giemsa staining in NUF2-KD 8505C group (Second row, right picture) in Fig. S2A. The corrected supplementary information file and original data were uploaded as attachment below. In addition, we have checked the original data twice and found no new errors. We wonder if it is possible to publish an erratum to correct our mistake.

The region of interest chosen from original picture in NUF2-WT group.
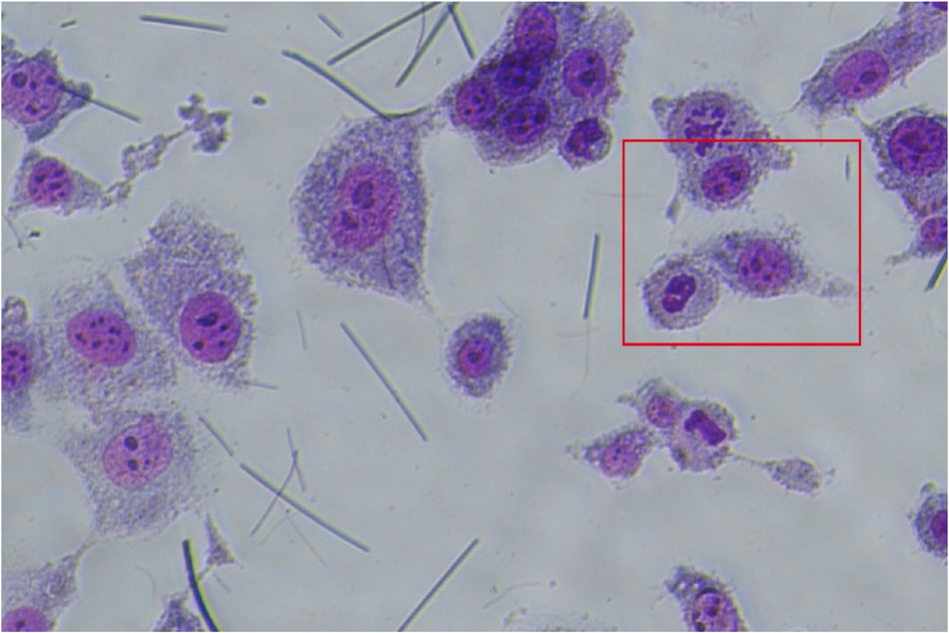


The region of interest chosen from original picture in NUF2-KD group.
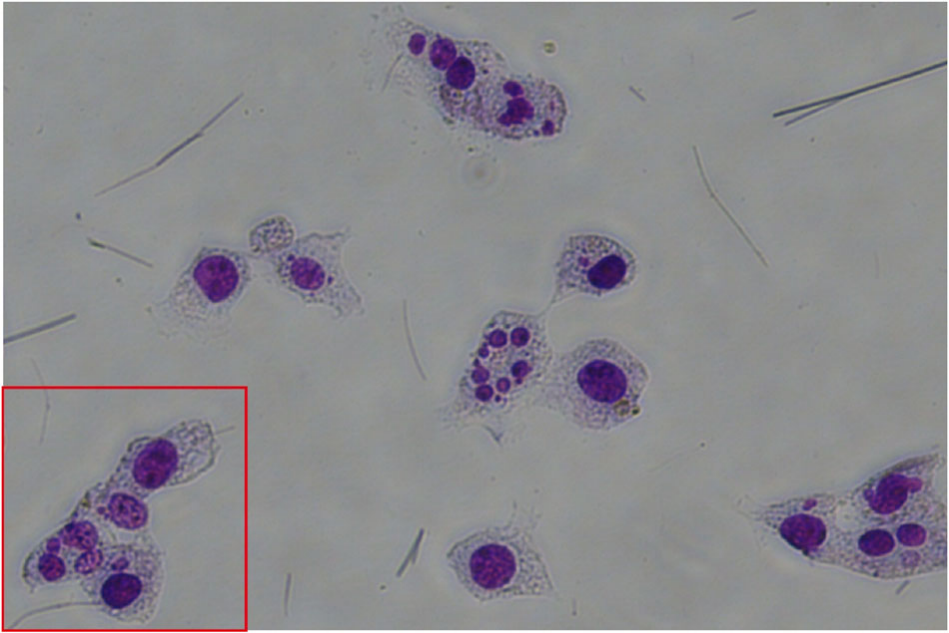


The primary figure S2.
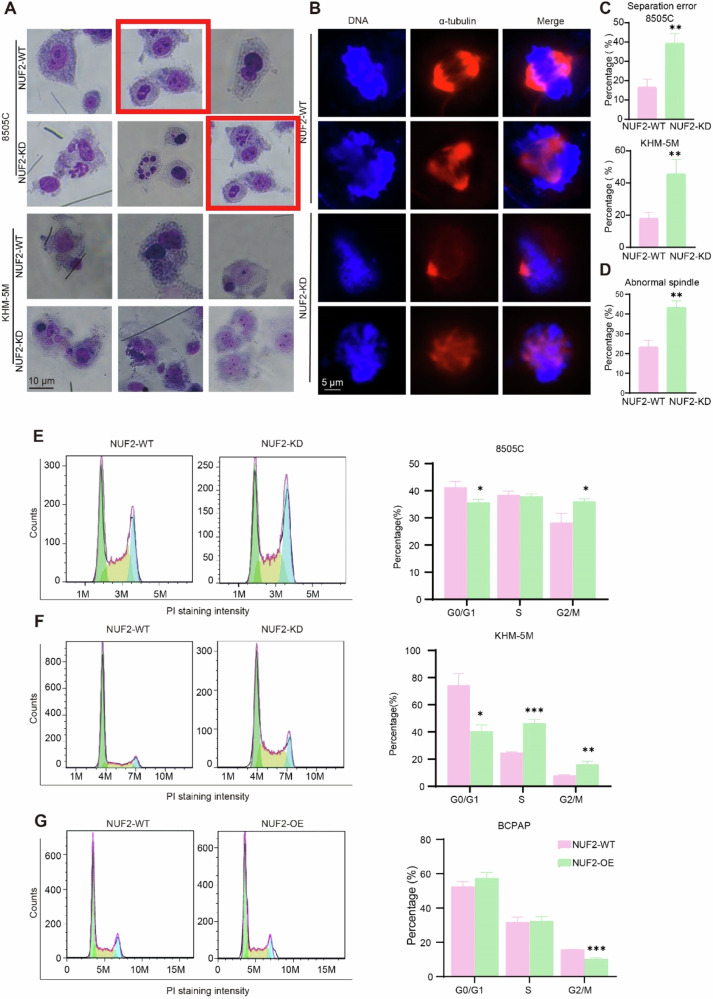


The correct Figure S2.
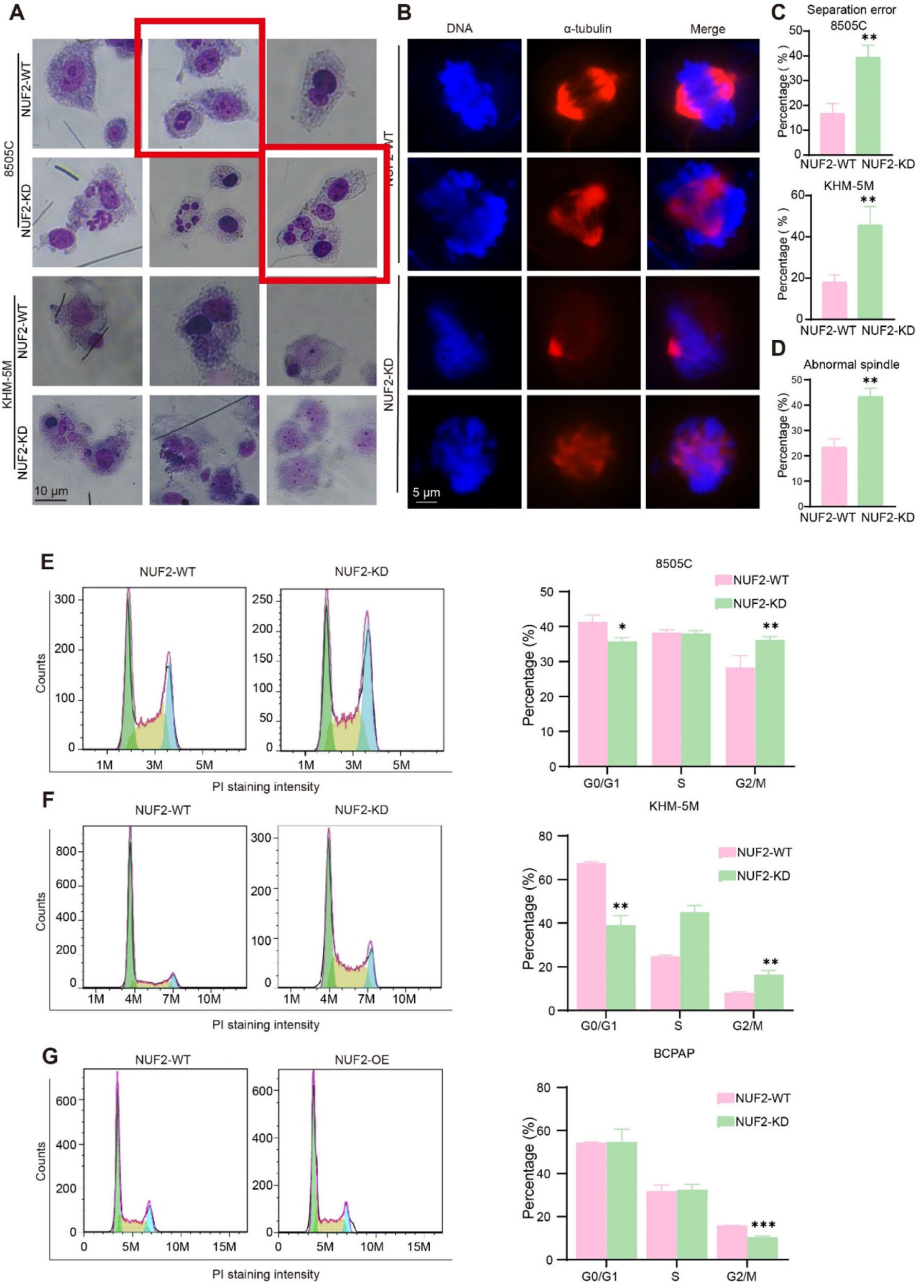


The original article has been corrected.

## Supplementary information

Figure S2

