# Correction: Maintenance of magnesium homeostasis by NUF2 promotes protein synthesis and anaplastic thyroid cancer progression

**DOI:** 10.1038/s41419-025-07348-y

**Published:** 2025-03-11

**Authors:** Lisha Bao, Yingying Gong, Yulu Che, Ying Li, Tong Xu, Jinming Chen, Shanshan Wang, Zhuo Tan, Ping Huang, Zongfu Pan, Minghua Ge

**Affiliations:** 1 Otolaryngology & Head and Neck Center, Cancer Center, Department of Head and Neck Surgery, Zhejiang Provincial People’s Hospital (Affiliated People’s Hospital), Hangzhou Medical College, Hangzhou, Zhejiang China; 2 Clinical Pharmacy Center, Department of Pharmacy, Zhejiang Provincial People’s Hospital (Affiliated People’s Hospital), Hangzhou Medical College, Hangzhou, China; 3 Zhejiang Key Laboratory of Precision Medicine Research on Head & Neck Cancer, Hangzhou, China; 4 Zhejiang Provincial Clinical Research Center for malignant tumor, Hangzhou, China

**Keywords:** Endocrine cancer, Oncogenes

Correction to: *Cell Death & Disease* 10.1038/s41419-024-07041-6, published online 06 September 2024

In this article in Fig. 3I, the image of NUF2-WT mice on day 10 (First row, second column) has been updated as well as the image of NUF2-KD mice on day 13 (Second row, third column).

**Incorrect Figure 3** 
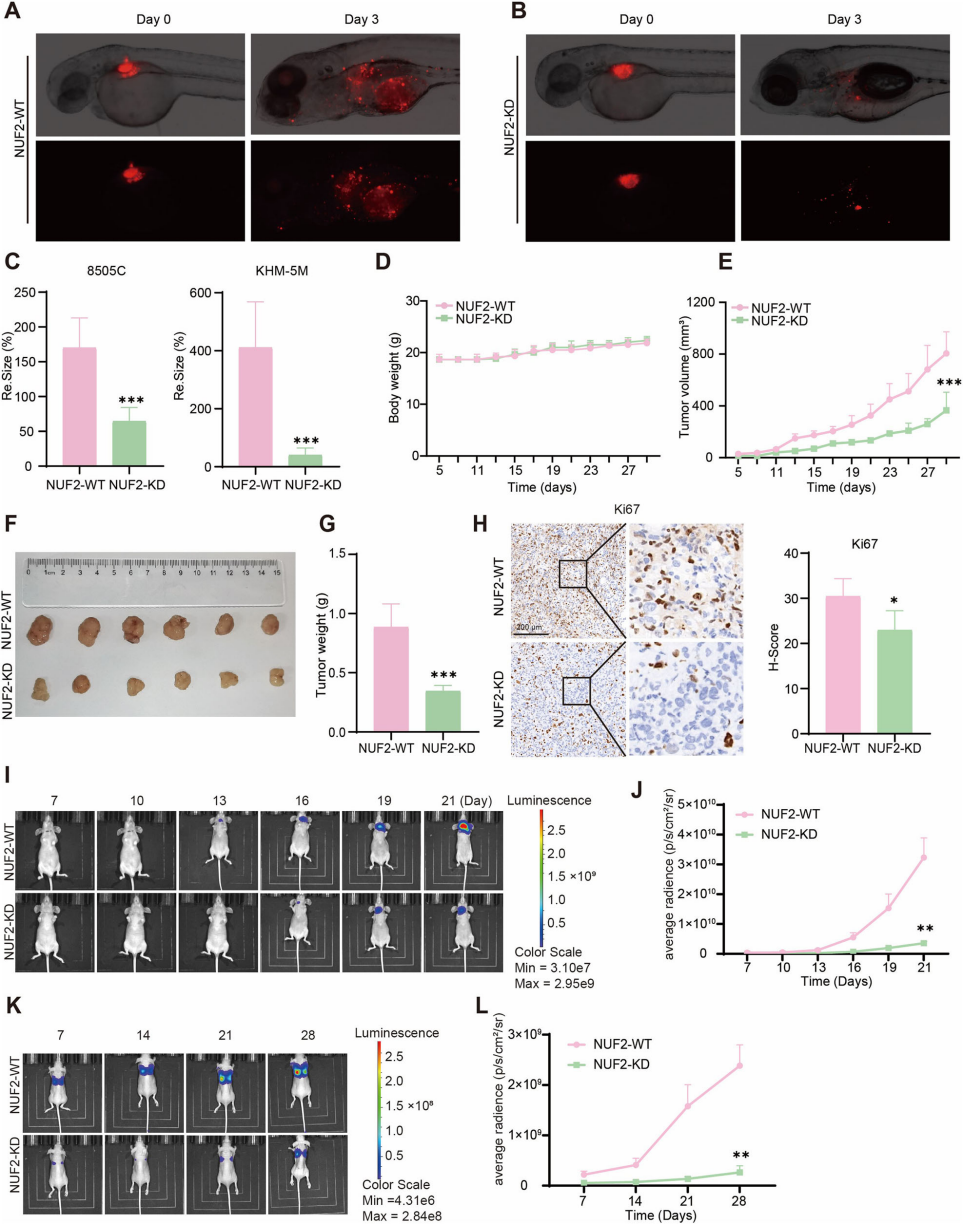


**Correct Figure 3** 
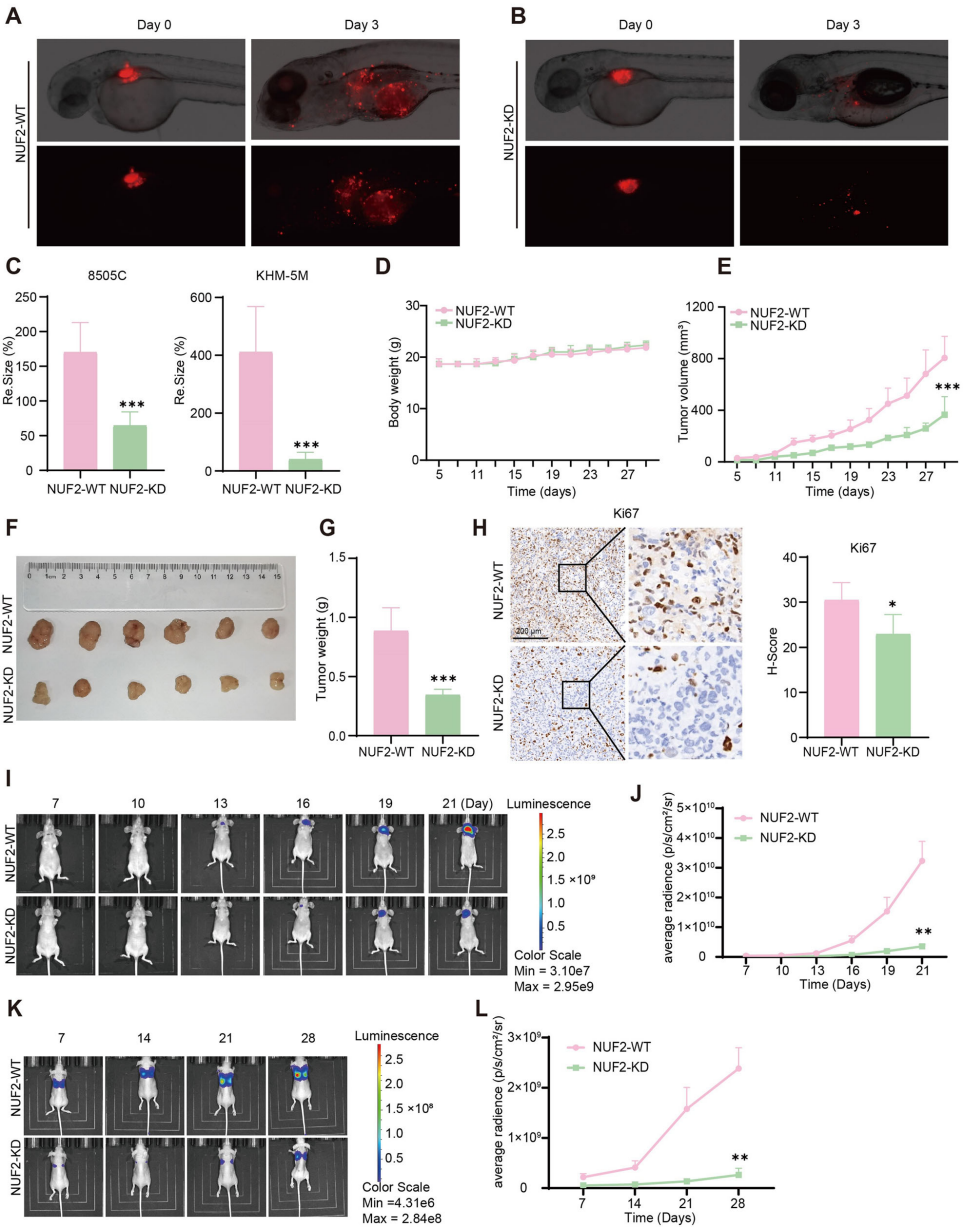


In Fig. 6C, the merge image of Nthy-ori 3-1 (First row, fourth column) has been updated.

**Incorrect Figure 6** 
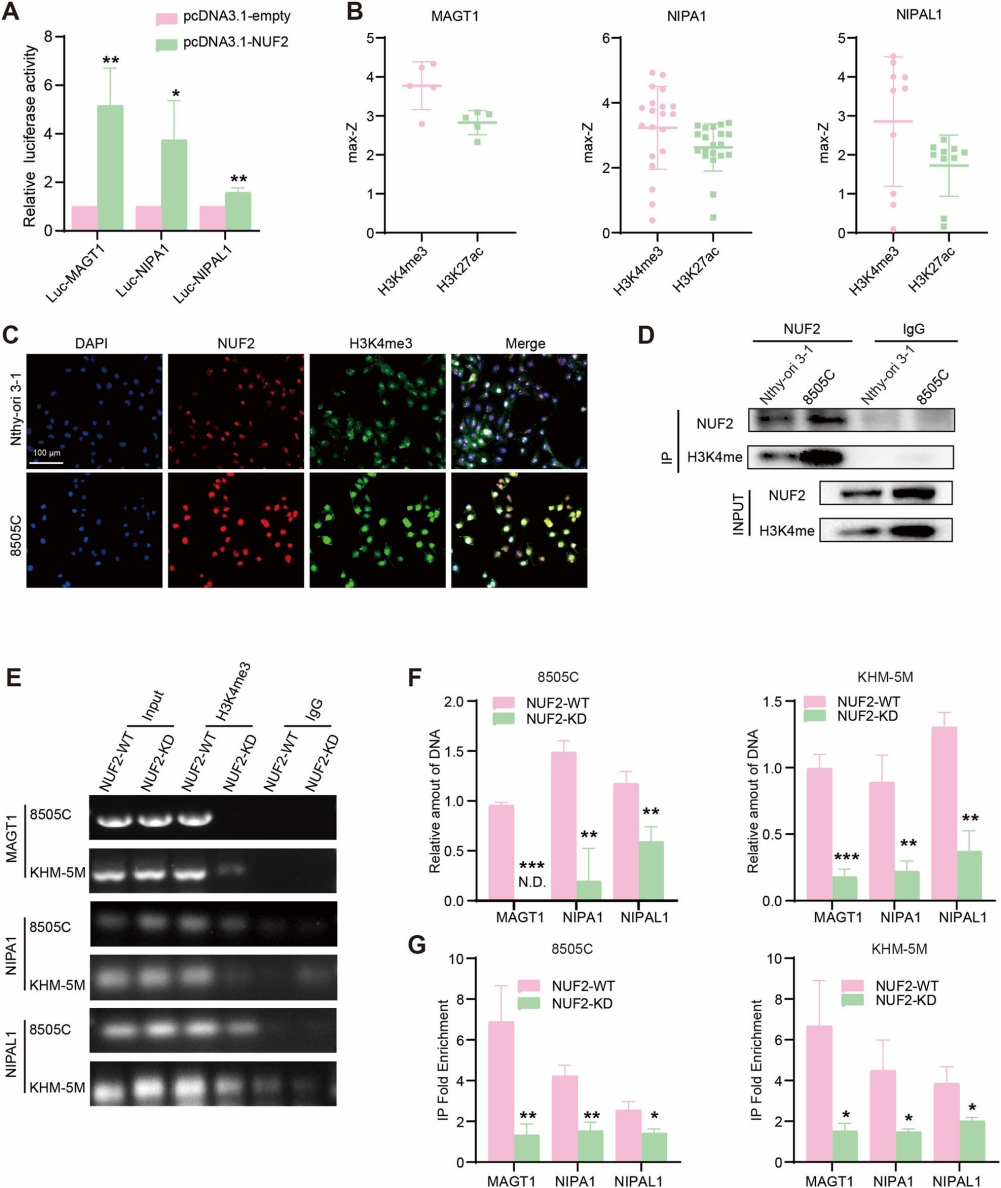


**Correct Figure 6** 
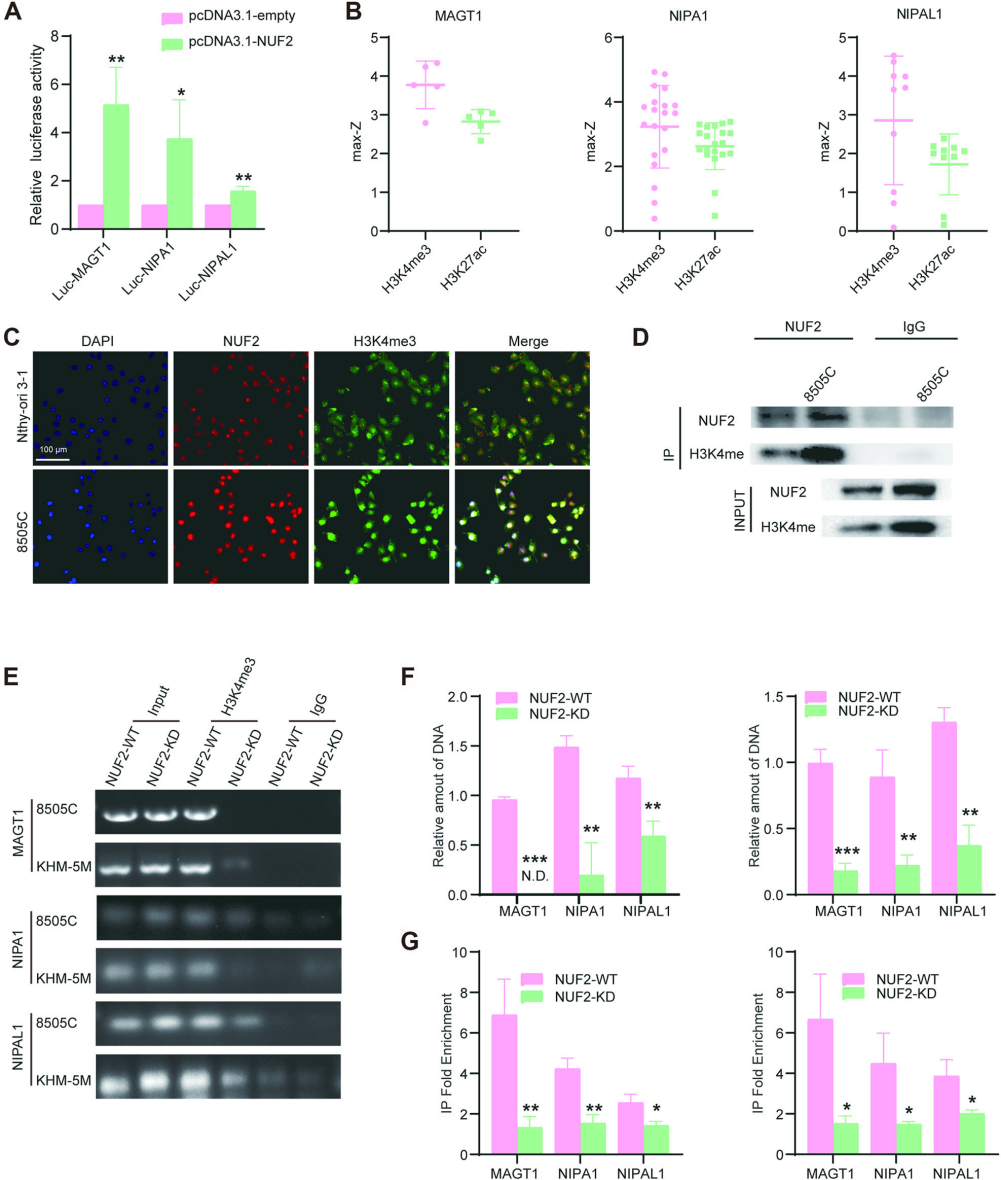


The animal experiments are conducted using six mice per group and imaged for six time points. The immunofluorescence staining for NUF2 and H3K4me3 in 8505C and Nthy-ori 3-1 cell were three replicates in each group. While we believe these errors did not affect the conclusions of our research, we deeply regret our oversight and sincerely apologize for the inconvenience caused.

The original article has been corrected.

