# Supplementary material for: Maintenance of magnesium homeostasis by NUF2 promotes protein synthesis and anaplastic thyroid cancer progression
Source: Cell Death Dis. 2024 Sep 6;15(9):656. doi: 10.1038/s41419-024-07041-6 (PMC11379715; doi:10.1038/s41419-024-07041-6)
Supplement: Supplementary file 1 — Supplementary Material [file 41419_2024_7041_MOESM1_ESM.docx]

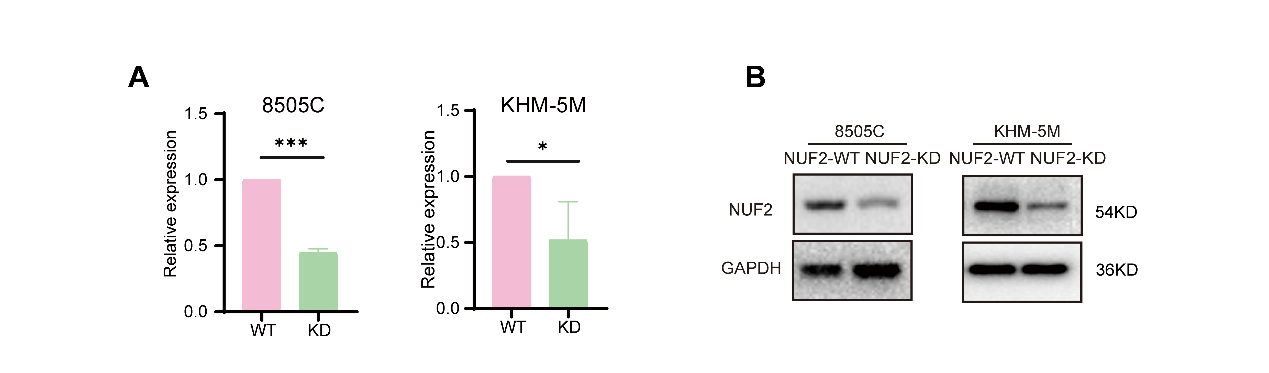


**Fig.S1. The knockdown efficiency of NUF2 in ATC cells analyzed by q-PCR and western blot.**


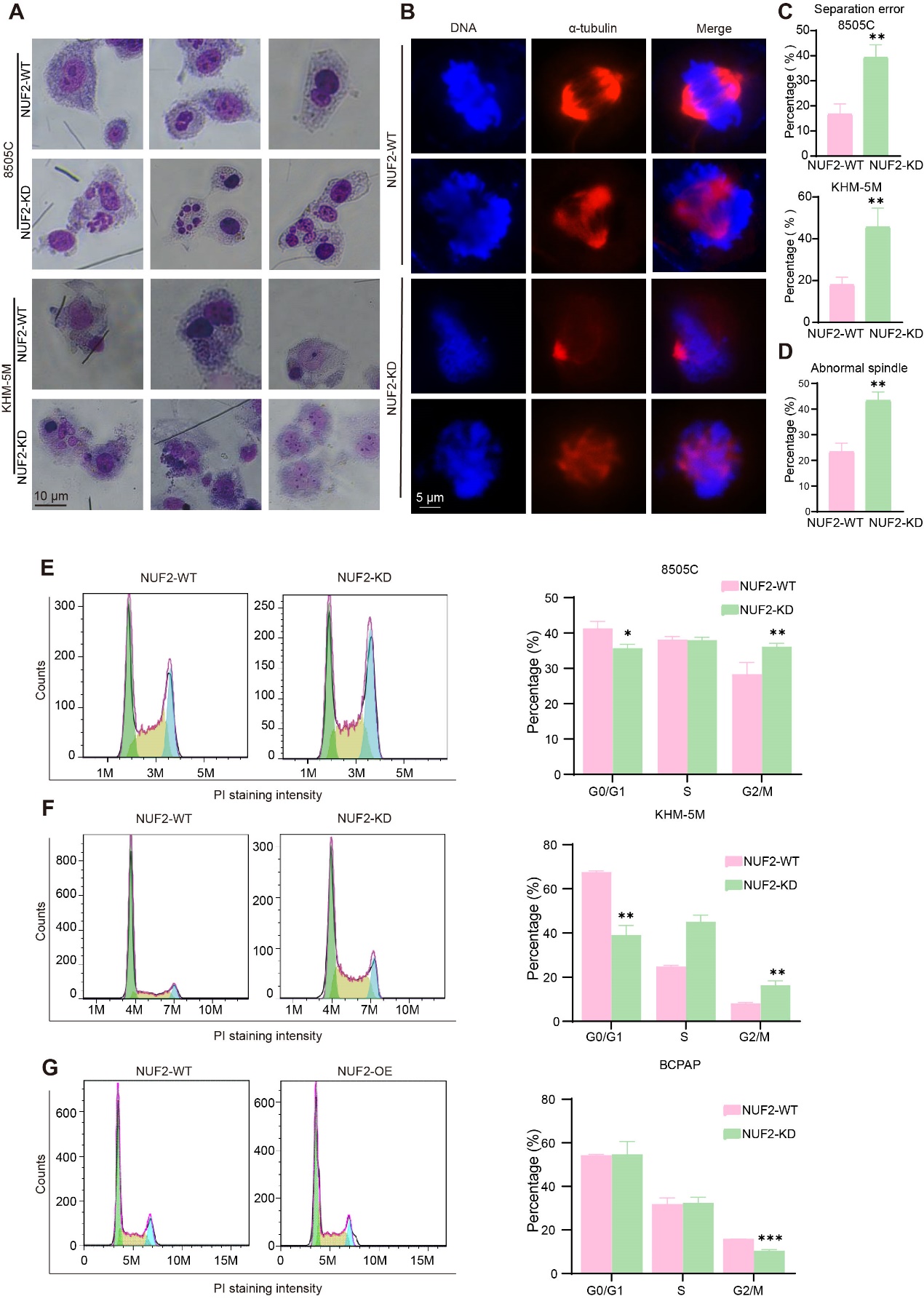


**Fig.S2. NUF2 involved in cell division (A)** Giemsa staining of ATC cells after NUF2 silence. **(B)** Tubulin IF staining of ATC cells after NUF2 silence. **(C-D)** Separation error percentages and abnormal spindle percentages of NUF2-WT and NUF2-KD ATC cells. **(E-G)** Cell cycle of ATC cells transfected with siRNA-NUF2 or siRNA-NC. **(H)** Cell cycle of NUF2-WT and NUF2-OE BCPAP cells. **(G)** The expression of NUF2 in different cell cycle phases.


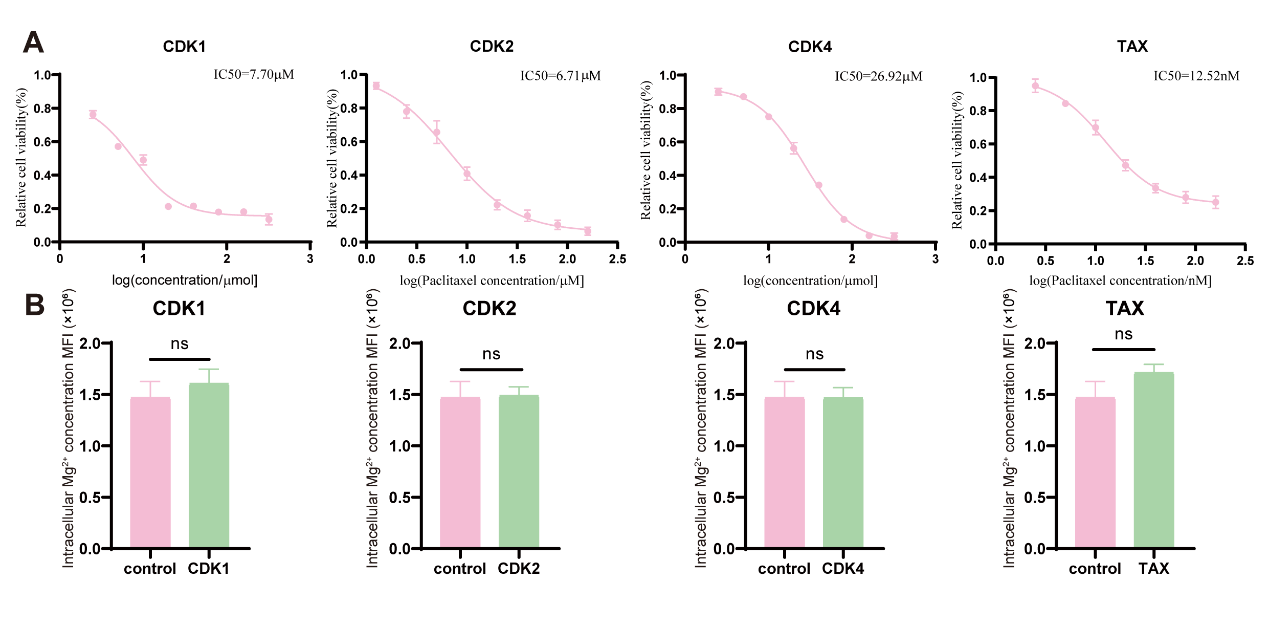


**Fig.S3. Expression of NUF2 in ATC and clinical relevance. (A)** The half maximal inhibitory concentration (IC50) of cell cycle inhibitor to 8505C cells. **(B)** The intracellular magnesium ion concentration in cancer cells treated with of cell cycle inhibitor.
