# Supplementary figures and images for: Maintenance of magnesium homeostasis by NUF2 promotes protein synthesis and anaplastic thyroid cancer progression
Source: Cell Death Dis. 2024 Sep 6;15(9):656. doi: 10.1038/s41419-024-07041-6 (PMC11379715; doi:10.1038/s41419-024-07041-6)

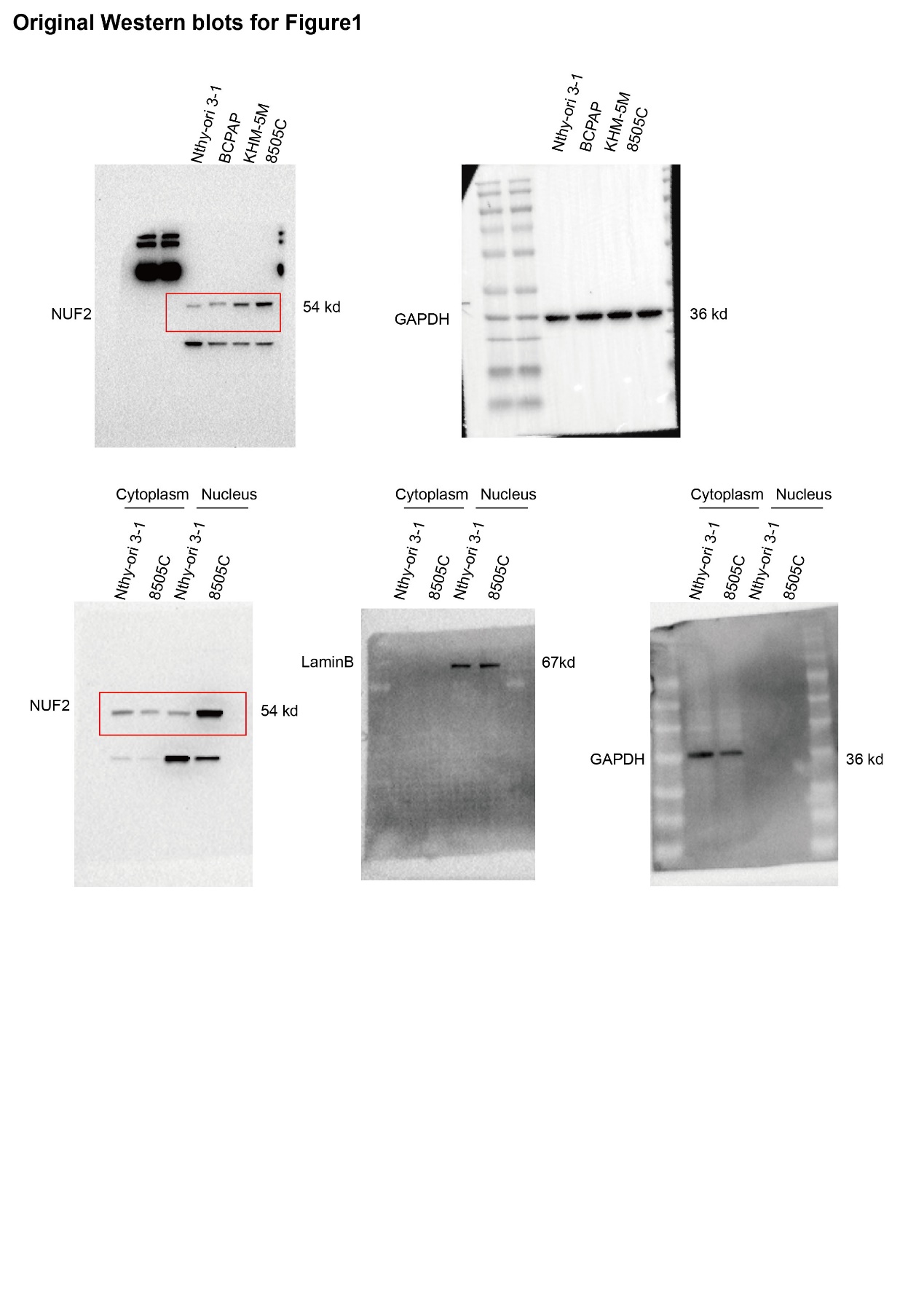

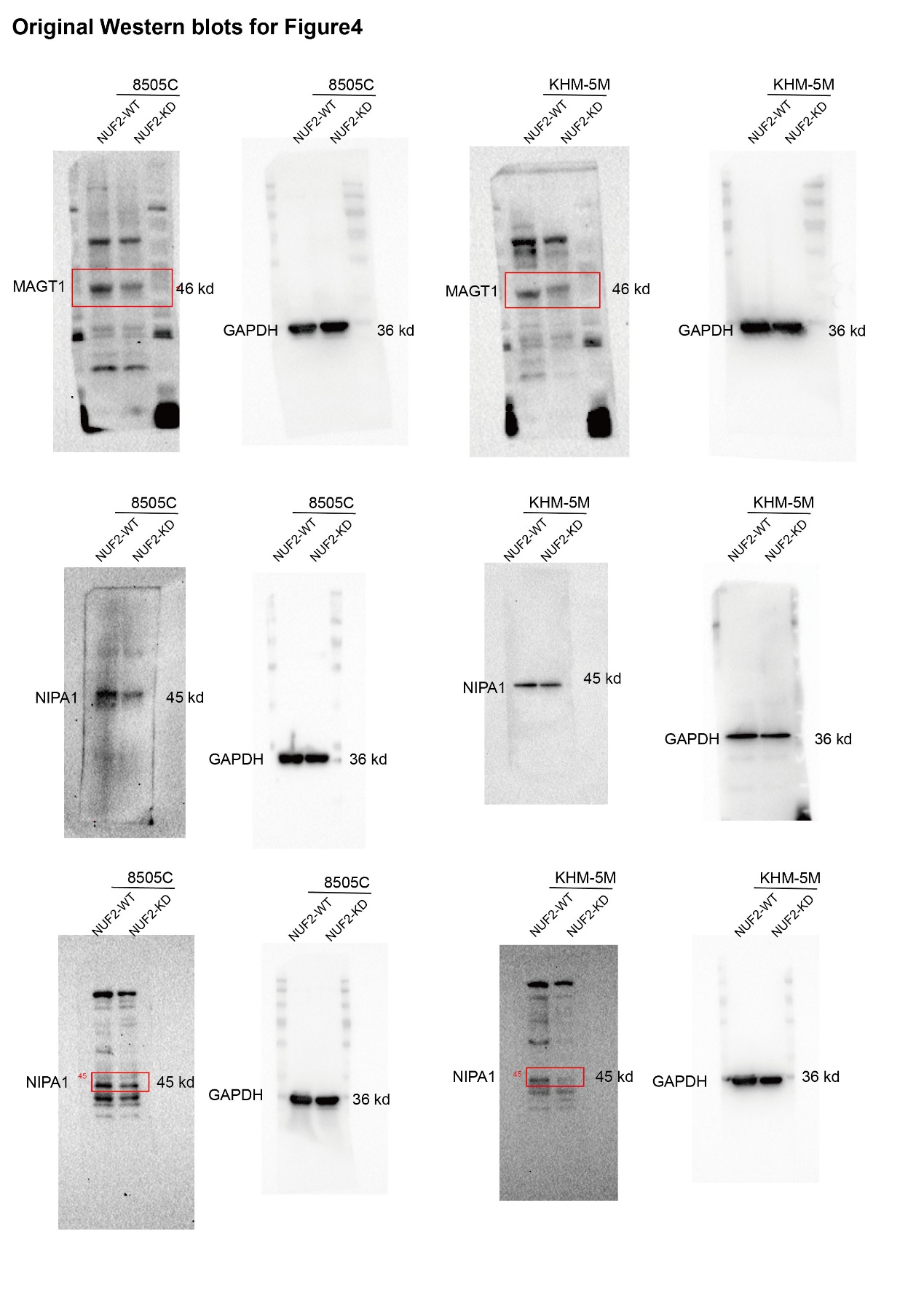

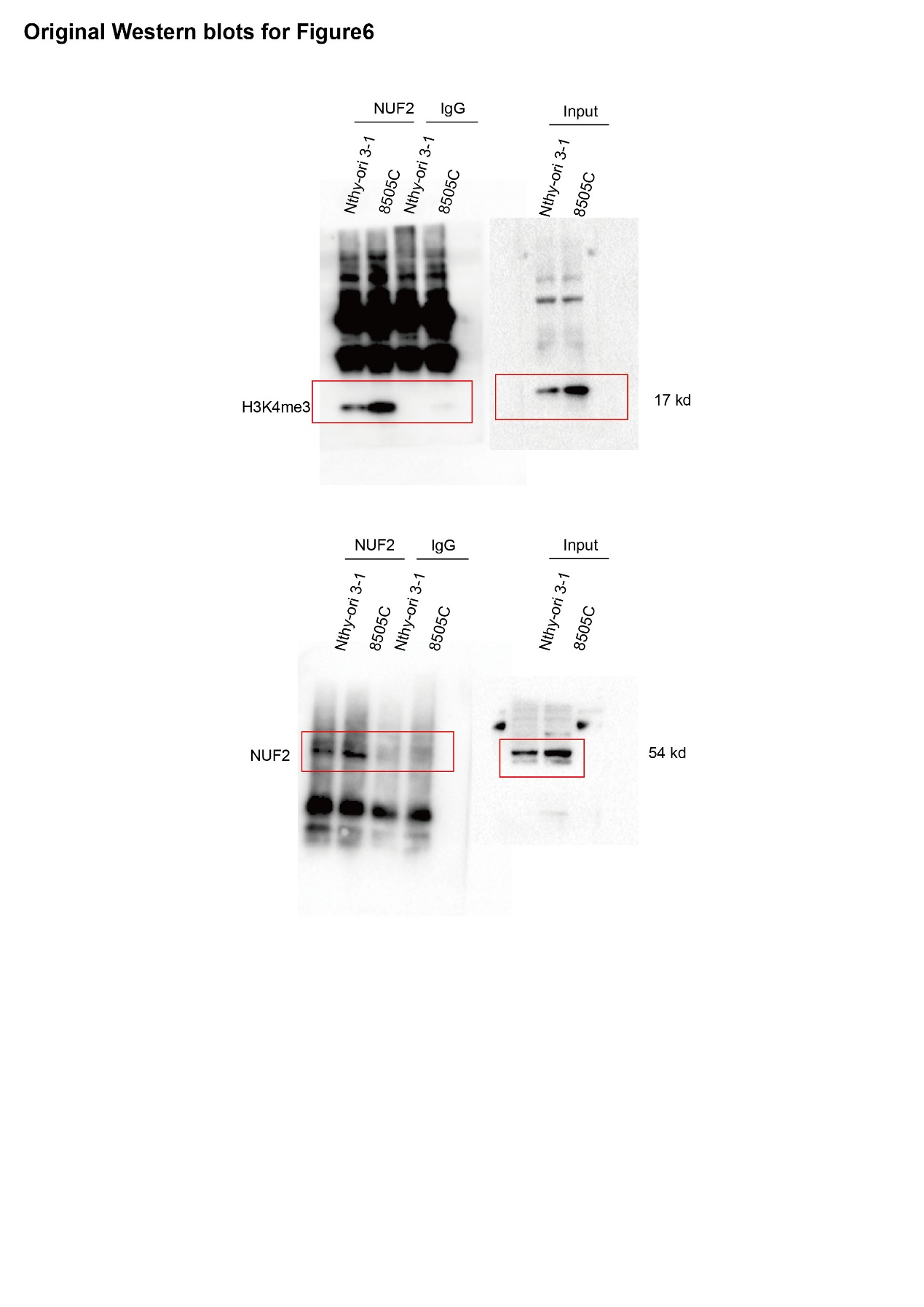

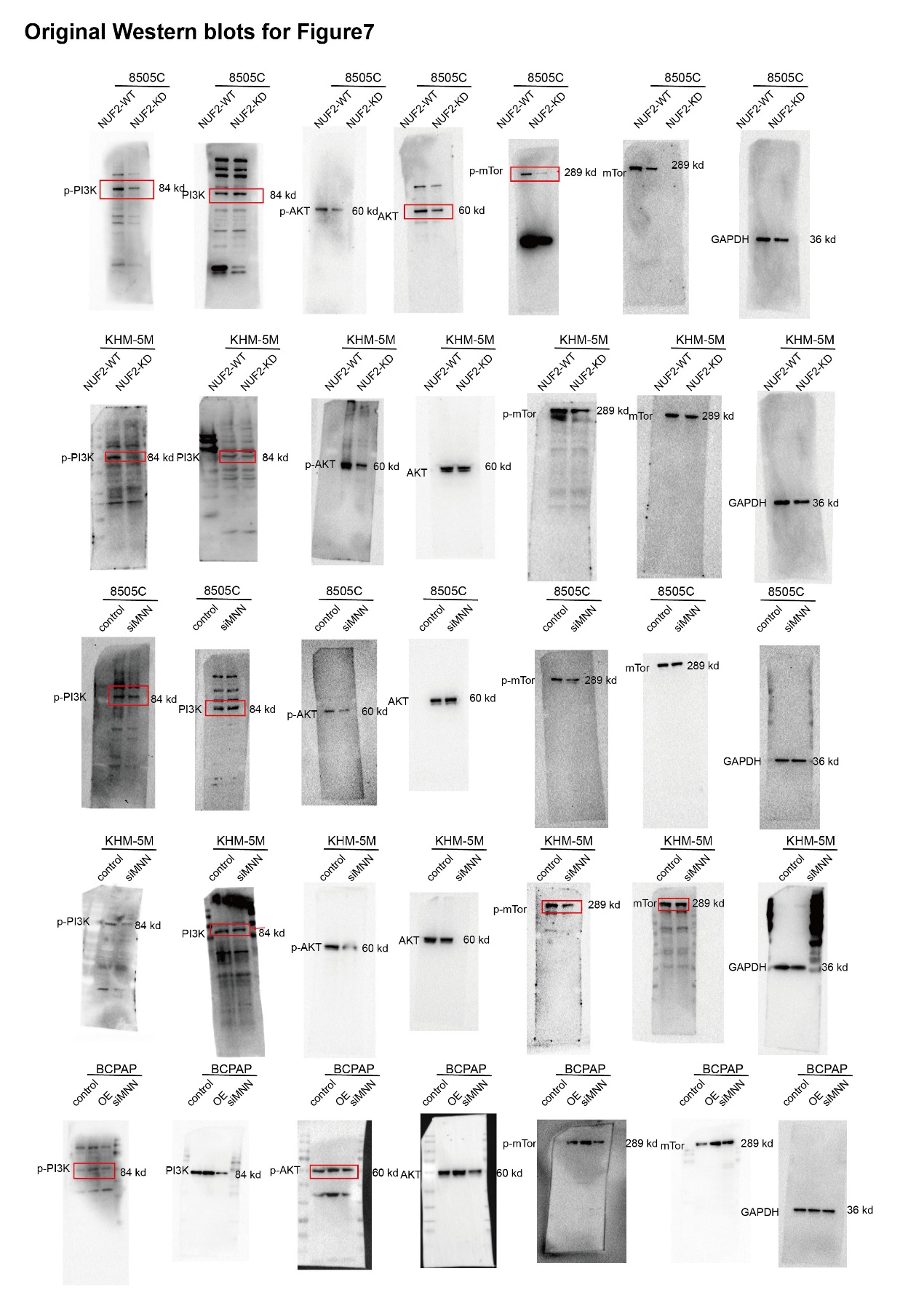

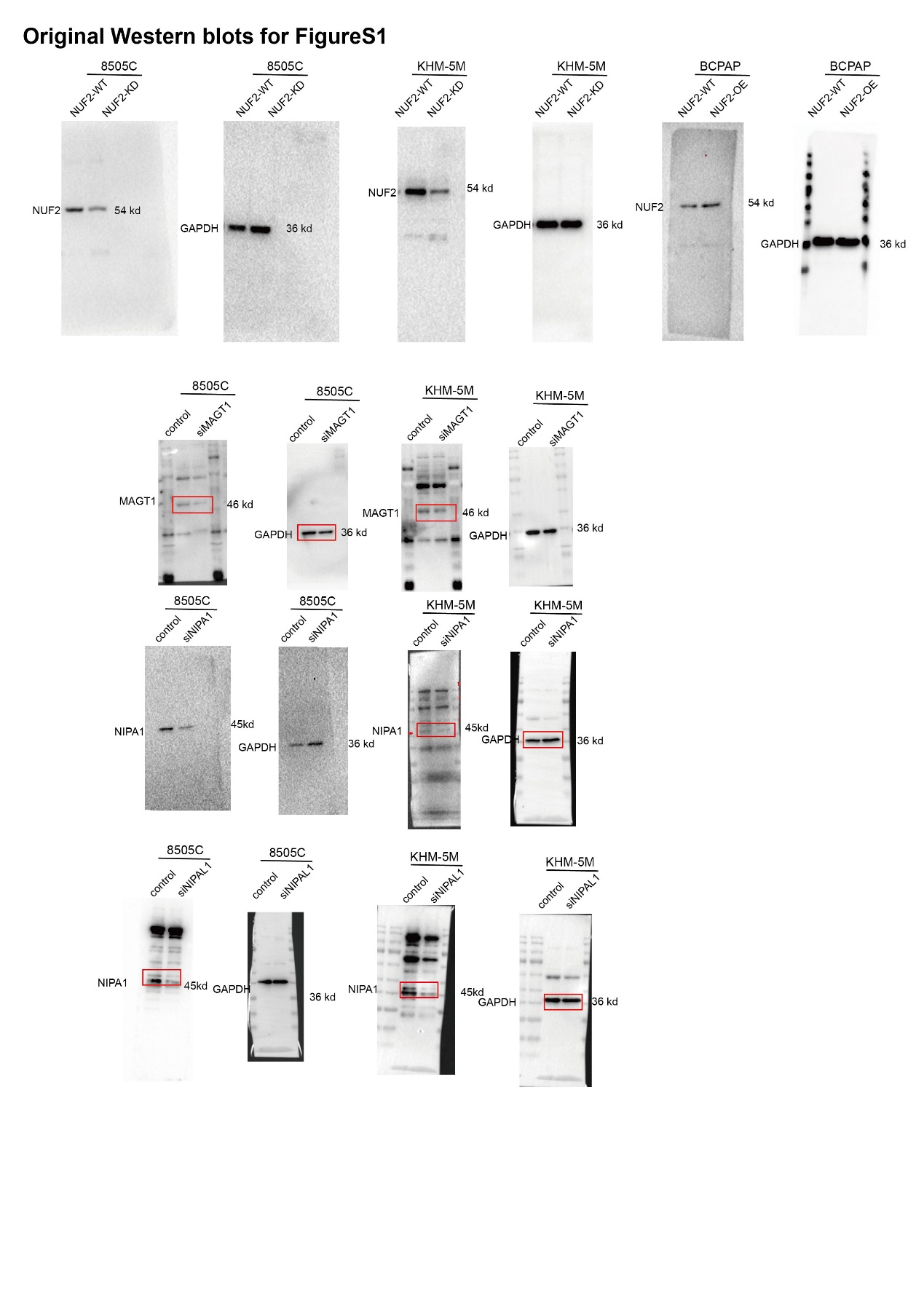

Supplement: Supplementary file 2 — Orinigal Western blot [file 41419_2024_7041_MOESM2_ESM.docx]
