# Supplementary figures and images for: Correction: Maintenance of magnesium homeostasis by NUF2 promotes protein synthesis and anaplastic thyroid cancer progression
Source: Cell Death Dis. 2024 Nov 18;15(11):842. doi: 10.1038/s41419-024-07174-8 (PMC11574243; doi:10.1038/s41419-024-07174-8)

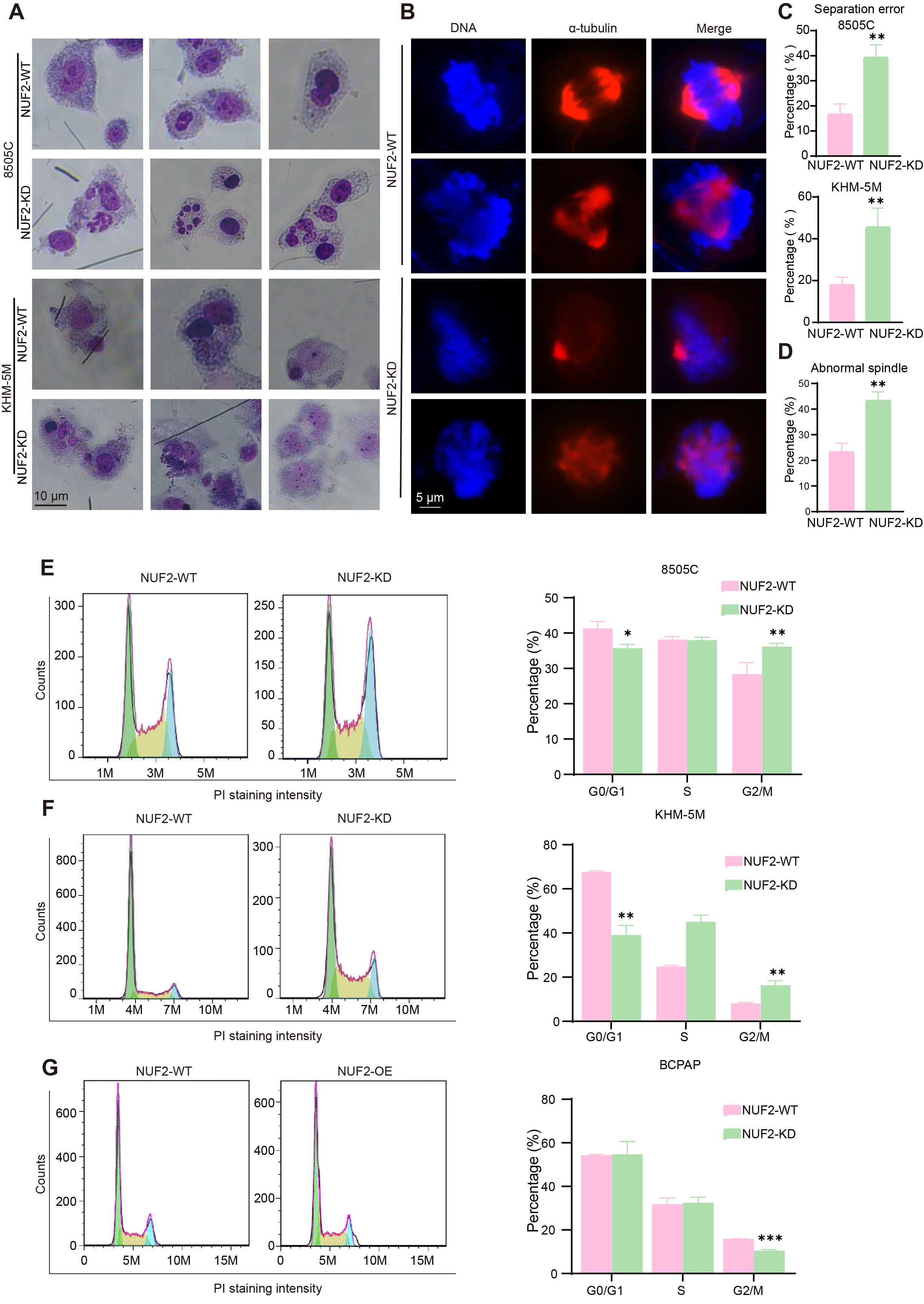

Supplement: Supplementary file 1 — Figure S2 [file 41419_2024_7174_MOESM1_ESM.jpg]
